# Deficiency of Mitochondrial Aspartate-Glutamate Carrier 1 Leads to Oligodendrocyte Precursor Cell Proliferation Defects Both In Vitro and In Vivo

**DOI:** 10.3390/ijms20184486

**Published:** 2019-09-11

**Authors:** Sabrina Petralla, Luis Emiliano Peña-Altamira, Eleonora Poeta, Francesca Massenzio, Marco Virgili, Simona Nicole Barile, Luigi Sbano, Emanuela Profilo, Mariangela Corricelli, Alberto Danese, Carlotta Giorgi, Rita Ostan, Miriam Capri, Paolo Pinton, Ferdinando Palmieri, Francesco Massimo Lasorsa, Barbara Monti

**Affiliations:** 1 Department of Pharmacy and BioTechnology, University of Bologna, 40126 Bologna, Italy; sabrina.petralla2@unibo.it (S.P.); luis_emiliano.pena_altamira@kcl.ac.uk (L.E.P.-A.); eleonora.poeta3@unibo.it (E.P.); francesca.massenzio@kcl.ac.uk (F.M.); marco.virgili@unibo.it (M.V.); 2 Department of Biosciences, Biotechnologies and Biopharmaceutics, University of Bari, 70121 Bari, Italyemanuela.profilo@hotmail.it (E.P.); ferdpalmieri@gmail.com (F.P.); 3 Department of Morphology, Surgery and Experimental Medicine, Section of Pathology, Oncology and Experimental Biology, Laboratory for Technologies of Advanced Therapies (LTTA), University of Ferrara, 44121 Ferrara, Italy; sbnlgu@unife.it (L.S.); crrmng@unife.it (M.C.); dnslrt@unife.it (A.D.); grgclt@unife.it (C.G.); paolo.pinton@unife.it (P.P.); 4 Department of Experimental, Diagnostic and Specialty Medicine (DIMES, Dipartimento di Medicina Specialistica Diagnostica e Sperimentale) and C.I.G. Interdepartmental Centre “L. Galvani”, University of Bologna, 40126 Bologna, Italy; rita.ostan3@unibo.it (R.O.); miriam.capri@unibo.it (M.C.); 5 Maria Cecilia Hospital, GVM Care & Research, Cotignola, 48010 Ravenna, Italy; 6 Institute of Biomembranes, Bioenergetics and Molecular Biotechnologies IBIOM, CNR, 70126 Bari, Italy

**Keywords:** mouse model, growth factors, subventricular zone, AGC1 deficiency, mitochondrial disease

## Abstract

Aspartate-Glutamate Carrier 1 (AGC1) deficiency is a rare neurological disease caused by mutations in the solute carrier family 25, member 12 (*SLC25A12*) gene, encoding for the mitochondrial aspartate-glutamate carrier isoform 1 (AGC1), a component of the malate–aspartate NADH shuttle (MAS), expressed in excitable tissues only. AGC1 deficiency patients are children showing severe hypotonia, arrested psychomotor development, seizures and global hypomyelination. While the effect of AGC1 deficiency in neurons and neuronal function has been deeply studied, little is known about oligodendrocytes and their precursors, the brain cells involved in myelination. Here we studied the effect of AGC1 down-regulation on oligodendrocyte precursor cells (OPCs), using both in vitro and in vivo mouse disease models. In the cell model, we showed that a reduced expression of AGC1 induces a deficit of OPC proliferation leading to their spontaneous and precocious differentiation into oligodendrocytes. Interestingly, this effect seems to be related to a dysregulation in the expression of trophic factors and receptors involved in OPC proliferation/differentiation, such as Platelet-Derived Growth Factor α (PDGFα) and Transforming Growth Factor βs (TGFβs). We also confirmed the OPC reduction in vivo in AGC1-deficent mice, as well as a proliferation deficit in neurospheres from the Subventricular Zone (SVZ) of these animals, thus indicating that AGC1 reduction could affect the proliferation of different brain precursor cells. These data clearly show that AGC1 impairment alters myelination not only by acting on N-acetyl-aspartate production in neurons but also on OPC proliferation and suggest new potential therapeutic targets for the treatment of AGC1 deficiency.

## 1. Introduction

Aspartate-glutamate carrier 1 (AGC1) deficiency is a rare neurological disease caused by mutations in the solute carrier family 25 member 12 (*SLC25A12*) gene, encoding for the mitochondrial aspartate-glutamate carrier isoform 1 (AGC1) [1,2,3]. AGC1 catalyses the unidirectional exchange between intra-mitochondrial aspartate and cytosolic glutamate and is a component of the malate–aspartate shuttle, thus playing a crucial role in the transfer of reducing NADH equivalents from the cytosol to mitochondria [4,5]. Interestingly, AGC1 is highly expressed in excitable tissues, such as muscle and brain [6]. By contrast, the second isoform of the mitochondrial aspartate/glutamate carrier, AGC2 [5], encoded by the *SLC25A13* gene, is mainly expressed in liver and mutations in this gene lead to neonatal intrahepatic cholestasis caused by citrin deficiency (NICCD) and adult-onset type II citrullinemia (CTLN2) [7].

AGC1 deficiency is a recently identified infantile epileptic encephalopathy (EIEE39, OMIM 612949) characterized by severe hypotonia, arrested psychomotor development and global cerebral hypomyelination, that manifests when AGC1 activity is completely abolished [1] or even drastically reduced [2]. A common feature of all patients is the marked reduction in brain levels of N-Acetyl-Aspartate (NAA), which is a crucial metabolite for myelin formation in the Central Nervous System (CNS) [8]. A similar defect in NAA production was also observed in AGC1-deficient mice, further confirming the importance of this mitochondrial carrier in myelination [9]. Many studies have been carried out in the same animal model to evaluate the effect of AGC1 deficiency in neuronal maturation and activity, showing that AGC1 plays an important role in cortical axon generation, postnatal development of cortico-hippocampal neurons, the nigrostriatal dopaminergic system and in the visual system, including the retina [10,11,12,13,14,15]. However, little attention has been given to oligodendrocytes, which are the most relevant brain cells involved in myelination. In this context, it has been observed that O4-positive cells (which include late precursors and immature premyelinating oligodendrocytes) are present in AGC1-deficient mice, though they present a different morphology, thus suggesting a change in their maturation [10]. Oligodendrocytes derive from oligodendrocyte precursor cells (OPCs), which continuously proliferate and differentiate into oligodendrocytes when the latter are needed to increase myelination during development and remyelination in the adult brain. Failure in the remyelination process leads to demyelinating diseases and OPC proliferation and differentiation are critical for spontaneous remyelination [16,17]. Indeed, primary OPCs with 60% down-regulated AGC1 displayed reduced myelin basic protein (MBP) expression, suggesting an oligodendrocyte-autonomous effect of AGC1 on myelination [18].

Here we studied the effect of AGC1 impairment on OPCs thoroughly, by using both in vitro and in vivo models. Our in vitro cell model is represented by Oli-Neu stable cell clones, which are immortalized mouse OPCs where a partial silencing of the *SLC25A12* gene was obtained by using a specific shRNA. Through this approach, we previously obtained stable cell lines of Neuro2A cells, in which we demonstrated that AGC1 impairment is associated with reduced proliferation and low NAA levels in undifferentiated neurons [19]. Our in vivo model is represented by C57BL/6N AGC1^+/−^ mice generated through the targeting of a 6.5 kb VICTR 76 construct into intron 2-3 of the *SLC25A12* mouse gene. In both models, as well as in neurospheres derived from the mouse subventricular zone (SVZ), we focused on OPC differentiation and proliferation and demonstrated that AGC1 down-regulation reduces OPC proliferation through the dysregulation of biochemical pathways involving trophic factors, such as PDGFα and TGFβs.

## 2. Results

### 2.1. Effect of AGC1 Silencing on Oli-Neu Cell Differentiation and Proliferation

**Figure 1:**
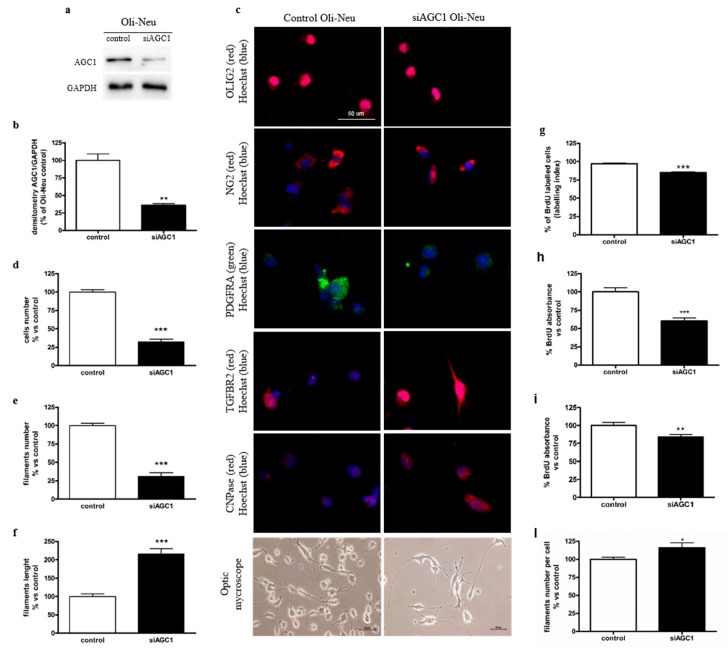
Spontaneous oligodendrocyte precursor cell (OPC) differentiation and OPC proliferation defects in aspartate glutamate carrier 1 (AGC1)-silenced Oli-Neu cells. Western blot analysis (**a**) and relative densitometries (**b**) of AGC1 expression in Oli-Neu cells, in which a partial silencing of the mouse AGC1 gene has been produced (siAGC1). Densitometry is the *ratio* between the expression level of AGC1 and GAPDH (Glyceraldehyde 3-phosphate dehydrogenase) as reference loading control and is expressed as percentage vs. control Oli-Neu cells. Immunofluorescence staining and optical microscopy images (**c**) of control and siAGC1 Oli-Neu cells. Nuclei were labelled with Hoechst, while Olig2, NG2, PDGFαR, TGFβR2 and CNPase were used as specific markers for Oli-Neu cells. Analyses for cells number (**d**), total filaments number (**e**), filaments length (**f**) and filaments number per cell (l) calculated with Fiji ImageJ2 software. Scale bar: 50 μm. BrdU immunofluorescence of control and siAGC1 Oli-Neu cells with nuclei staining with Hoechst (blue) and BrdU positive-cell count analysis expressed as labelling index (**g**). BrdU incorporation by ELISA assay (enzyme-linked immunosorbent assay) in control and siAGC1 Oli-Neu cells after 6 (**h**) and 24 h (**i**) BrdU incubation. Scale bar: 100 μm. Values are the mean ± SE of 3 independent experiments performed in triplicate, * *p* < 0.05, ** *p* < 0.01, *** *p* < 0.001 compared to control Oli-Neu cells, Student’s *t*-test.

In order to study the effect of AGC1 impairment on oligodendrocyte precursor cells (OPCs), we produced stable clones of Oli-Neu cells (kindly provided by Dr. Jacky Trotter, University of Mainz, Germany) as a model of immortalized mouse OPCs, expressing a specific shRNA to down-regulate the AGC1 gene or a scrambled control sequence (see Materials and methods for further details). Western blots and densitometric analyses showed reduced AGC1 expression of about 70% in AGC1-silenced (siAGC1) Oli-Neu cells compared to control Oli-Neu cells (Figure 1a,b), an expression level that is comparable to the residual AGC1 activity observed in human patients [2]. We then analysed whether AGC1 silencing could affect Oli-Neu cell differentiation. We observed no difference in 1 mM db-cAMP-induced differentiation between control and siAGC1 Oli-Neu cells, including no change in the expression of myelin-associated glycoprotein (MAG) (Supplemental Figure S1a,b). However, analysis of cell filament length and number in non-stimulated siAGC1 Oli-Neu cells revealed a lower number, greater length of cell filaments and higher number of filaments per cell, as compared to control cells (Figure 1c–f,l), thus suggesting that Oli-Neu cells with down-regulated AGC1 are partially differentiated even in the absence of the db-cAMP stimulus.

Starting from this observation, that is, that AGC1 silencing seems to induce spontaneous differentiation of Oli-Neu cells, we decided to evaluate whether this effect could be related to a change in cell proliferation. Preliminary experiments with MTT assay and Trypan blue staining showed a slight but not significant difference in cell viability between control and siAGC1 Oli-Neu cells (data not shown). We thus performed cell counting 24 h after plating and we observed that siAGC1 Oli-Neu cell number was significantly lower compared to control Oli-Neu cells (Figure 1d). In order to understand whether reduced cell number depended on decreased proliferation, BrdU incorporation and subsequent immunofluorescence staining were performed showing significantly lower BrdU labelling in siAGC1 Oli-Neu cells compared to control cells (Figure 1g and Supplemental Figure S1c). We also quantified BrdU incorporation through ELISA after a 6-h (Figure 1h) and a 24-h (Figure 1i) BrdU pulse that confirmed the significant lower BrdU incorporation in siAGC1 Oli-Neu cells, strongly suggesting a significantly lower proliferation rate of OliNeu cells with impaired AGC1 activity as compared to control cells. In agreement with this, cell cycle analysis by flow cytometry showed that a slightly higher number of siAGC1 Oli-Neu cells were in G0/G1 phases compared to control Oli-Neu cells (64% vs. 58% respectively), as well as what observed for S phase (26% vs. 22% respectively) and G2/M phases (15% vs. 13% respectively, Supplemental Figure S2).

### 2.2. Effect of AGC1 Silencing on Oli-Neu Cell Lactate Production, Ros, [Ca^2+^], [Atp] and Mitochondrial Membrane Potential

**Figure 2:**
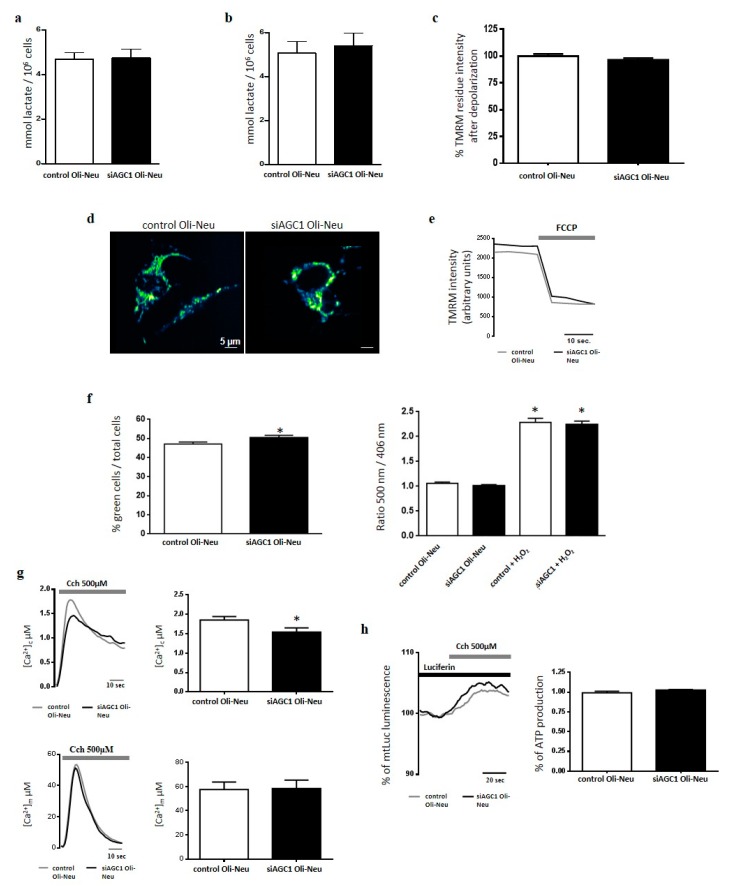
Effect of AGC1 down-regulation on lactic acid release, mitochondrial membrane potential, ROS generation and [Ca^2+^] homeostasis of Oli-Neu cells. Lactic acid was quantified in conditioned complete SATO mediumharvested from control Oli-Neu (white bars) or siAGC1-Oli-Neu cells (black bars) in the absence (**a**, undifferentiated cells) or presence (**b**, differentiated cells) of 1 mM dibutyryl-cAMP for 48 h. Values are the means ± SD from 3 independent experiments performed in triplicate. (**c**) Δψm was measured by fluorescence microscopy in control Oli-Neu (white bars) or siAGC1-Oli-Neu cells (black bars) incubated in minimal essential medium supplemented with 1 g/L glucose. Cells were loaded with 20 nM TMRM (tetramethyl rhodamine methyl ester) for 30 min at 37 °C and fluorescence intensities were imaged every 5 s with a fixed 20 milliseconds exposure time. Carbonyl cyanide-4-(trifluoromethoxy)phenylhydrazone (FCCP), an uncoupler of oxidative phosphorylation, was added after 12 acquisitions to completely collapse the electrical gradient established by the respiratory chain (**d**,**e**). Data are means ± SD of TMRM percentage intensities normalized to values before agonist stimulation in three independent experiments. (**f**) Cytosolic and mitochondrial hydrogen peroxide were measured in control-Oli-Neu (white bars) or siAGC1-Oli-Neu cells (black bars) loaded with 5 μM CM-H2DCFDA (left panel) or expressing the ratiometric H_2_O_2_-sensitive mt-HyPer protein (right panel). (**g**) Control-Oli-Neu (grey lines) or siGC1-OliNeu cells (black lines) expressing chimeric aequorins targeted to cytosol (upper panels) or mitochondria (lower panels) were perfused in KRB supplemented with glucose 1 g/L and stimulated with Carbachol 500 µM. Shown traces are representative of the following measurements: for control OliNeu cells, [Ca^2+^]c peak values, 1.54 ± 0.14 μM, *n* = 20; [Ca^2+^]m peak values, 58,4 ± 6.2 μM, *n* = 20; for siAGC1-OliNeu cells, [Ca^2+^]c peak values, 1.85 ± 0.09 μM, *n* = 20; [Ca^2+^]m peak values, 57,6 ± 4.1 μM, *n* = 20. (**h**) ATP-dependent luminescence was measured in control Oli-Neu (grey lines) or siAGC1-Oli-Neu cells (black lines) cells expressing the mitochondrially targeted luciferase (mtLuc) perfused in KRB supplemented with glucose 1 g/L and challenged with Carbachol 500 µM. Data are expressed as percentage of mtLuc light output increase from cells normalized to the prestimulatory values. Shown traces are representative of the following results: for control Oli-Neu cells, 101 ± 8%, *n* = 20 of the prestimulatory value; for siAGC1-OliNeu cells: 103 ± 9%, *n* = 20.

Since AGC1 down-regulation inhibited mitochondrial pyruvate oxidation in undifferentiated Neuro2A cells leading to increased production of lactic acid [19], we studied whether a similar effect occurred in siAGC1 Oli-Neu cells. After 48 h of incubation, lactic acid release in conditioned high-glucose medium from both undifferentiated and differentiated siAGC1 Oli-Neu cells was similar to that of conditioned media from control cells (Figure 2a,b). These data suggested that AGC1 down-regulation in Oli-Neu cells did not alter the rate of glucose oxidation. As shown by fluorescence microscopy, tetramethyl rhodamine methyl ester (TMRM)-stained mitochondria of siAGC1 Oli-Neu cells did not appear remarkably different from those of control cells in terms of morphology and fluorescence intensity (Figure 2c–e, Supplemental Figure S3), suggesting that AGC1 silencing in Oli-Neu cells did not affect the mitochondrial network and membrane potential. Furthermore, although siAGC1 Oli-Neu cells showed slight but significantly increased ROS production in the cytosol compared to control cells (Figure 2f), no difference in mitochondrial ROS levels were imaged with the ratiometric probe mt-Hyper (Figure 2g) used as an ROS sensitive probe directly targeted to mitochondria [20].

Considering that AGC1 is a Ca^2+^-stimulated transporter and its down-regulation in undifferentiated Neuro2A cells induced an increased mitochondrial response to Ca^2+^ stimulation [19], we tested whether a similar response also occurred in siAGC1 Oli-Neu cells challenged with Ca^2+^-releasing agonists. In siAGC1 Oli-Neu cells expressing the recombinant Ca^2+^-sensitive aequorin probe and stimulated with carbachol, cytosolic [Ca^2+^] ([Ca^2+^]_c_) was slightly but significantly increased when compared to control cells, though no difference in mitochondrial [Ca^2+^] ([Ca^2+^]_m_) was measured (Figure 2h). Consequently, in both types of cells expressing the recombinant luciferase targeted to mitochondria and stimulated with carbachol, the Ca^2+^-induced increase of mitochondrial ATP synthesis resulted unvaried (Figure 2i).

### 2.3. Effect of AGC1 Silencing on PDGFα and TGFβ Pathways in Oli-Neu Cells

**Figure 3:**
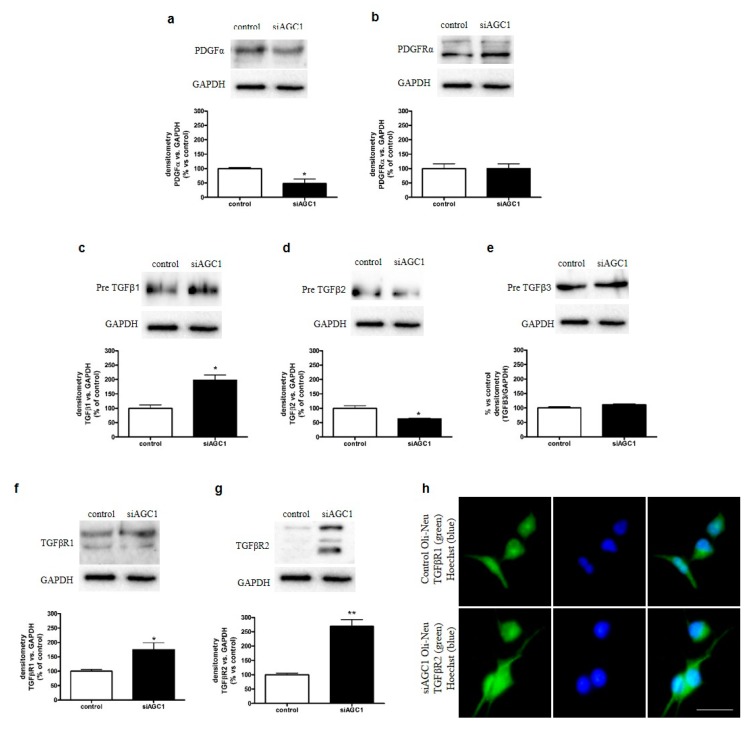
Dysregulation of Platelet-Derived Growth Factor α (PDGFα) and Transforming Growth Factor β (TGFβ) pathways in AGC1-silenced Oli-Neu cells. Western blot analysis and relative densitometries of PDGFα (**a**), PDGFαR (**b**), TGFβ1 (**c**), TGFβ2 (**d**), TGFβ3 (**e**), TGFβR1 (**f**) and TGFβR2 (**g**) expression in control and siAGC1 Oli-Neu cells. Densitometry is the *ratio* between the expression level of each protein and GAPDH as reference loading control and is expressed as percentage vs. control Oli-Neu cells. (**h**) Immunofluorescence staining of TGFβR1 in control and siAGC1 Oli-Neu cells (nuclei were labelled with Hoechst). Scale bar: 50 μm. Values are the mean ± SE of 3 independent experiments performed in triplicate, * *p* < 0.05, ** *p* < 0.01, compared to control Oli-Neu cells, Student’s *t*-test.

The absence of remarkable differences in mitochondrial function between control and siAGC1 Oli-Neu cells prompted us to investigate whether the observed difference in cell proliferation rates could depend on the alteration of trophic factors, in particular PDGFα and TGFβs, which are essential for OPC proliferation and differentiation, respectively. Through western blot analysis, we observed that siAGC1 Oli-Neu cells expressed lower PDGFα levels compared to control Oli-Neu cells (Figure 3a), whereas PDGFα receptor expression remained virtually the same (Figure 3b). Regarding the TGFβ pathways, only precursor TGFβs were expressed in Oli-Neu cells, whereas no mature (cleaved) forms were detected (Figure 3c–e). Pre-TGFβ1 expression was significantly higher in siAGC1 Oli-Neu cells compared to control Oli-Neu cells (Figure 3c). On the other hand, pre-TGFβ2 expression showed an opposite trend, its expression being lower (Figure 3d) in siAGC1 Oli-Neu cells compared to control cells, whereas no differences were detected for pre-TGFβ3 expression (Figure 3e). Expression levels of both TGFβ receptor 1 and 2 were also higher in siAGC1 Oli-Neu cells compared to control Oli-Neu cells (Figure 3f,g) and immunofluorescence analysis showed stronger labelling against TGFβ receptor 1 in siAGC1 Oli-Neu cells (Figure 3h). Overall, these data suggested that the expression of PDGFα, TGFβs and respective receptors, all known to play key roles in OPC proliferation and differentiation, is impaired in Oli-Neu cells with down-regulated AGC1.

### 2.4. Effects of TGFβ2 Treatment on Oli-Neu Cell Proliferation and Differentiation

**Figure 4:**
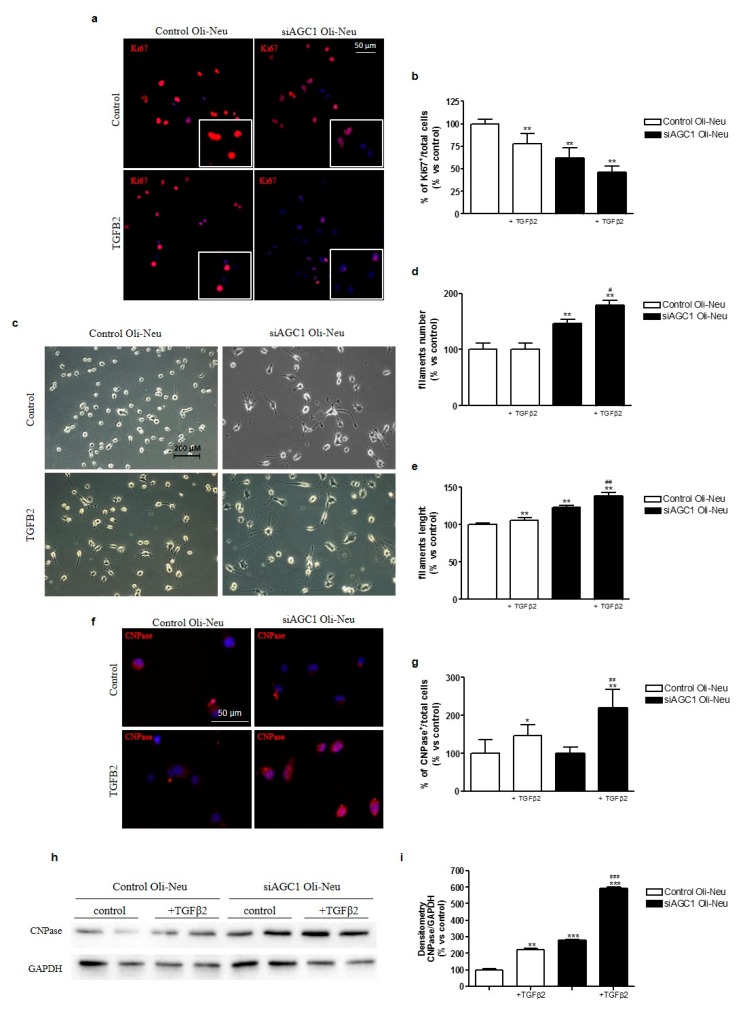
Effects of TGFβ2 treatment on control and AGC1-silenced Oli-Neu cells proliferation and differentiation. Immunofluorescence staining of Ki67 proliferation marker in control and siAGC1 Oli-Neu cells (nuclei were labelled with Hoechst) (**a**) and Ki67 positive-cell count analysis expressed as the *ratio* of Ki67 positive/Hoechst stained cells (**b**). Optical microscopy images (**c**) of control and siAGC1 Oli-Neu cells untreated and following treatment with TGFβ2. Scale bar: 200 μm. Filament number (**d**) and filament length (**e**) calculated by using Fiji ImageJ2 software (developed by the National Institutes of Health, NIH, USA). Immunofluorescence analysis of CNPase-positive cells (red, with Hoechst-labelled nuclei in blue) (**f**) and CNPase-positive cell count analysis respectively (**g**). Scale bar: 50 μm. Western blot analysis (**h**) and relative densitometries (**i**) of CNPase expression. Values are the mean ± SE of 3 independent experiments performed in triplicate, *p < 0.1, ** *p* < 0.01, *** *p* < 0.001 compared to control Oli-Neu cells, ## *p* < 0.01, ### *p* < 0.001 compared to siAGC1 Oli-Neu cells, Two-way ANOVA (Bonferroni’s post-test).

Based on the observed differences for pre-TGFβ2 and TGFβ receptor 2 expression in siAGC1 Oli-Neu cells compared to control cells, we investigated the activity of the TGFβ pathway by exposing Oli-Neu cells to exogenous TGFβ2 (10 ng/mL). After treatment for 24 h, TGFβ2 reduced proliferation in both control and siAGC1 Oli-Neu cells, as shown by Ki67 staining (Figure 4a,b) and increased cell differentiation, as demonstrated by the measurement of filament number and length, as well as by 2′,3′-Cyclic-nucleotide 3′-phosphodiesterase (CNPase) staining and expression (Figure 4c–i). Interestingly, this effect was more evident in siAGC1 Oli-Neu cells than in control cells, suggesting that the higher expression level of pre-TGFβ1 and TGFβ receptors could drive AGC1-impaired cells to become more sensitive to this differentiative stimulus.

### 2.5. OPC Proliferation and PDGFα/TGFβs Expression in AGC1^+/−^ Mice as an AGC1 Deficiency In Vivo Model

**Figure 5:**
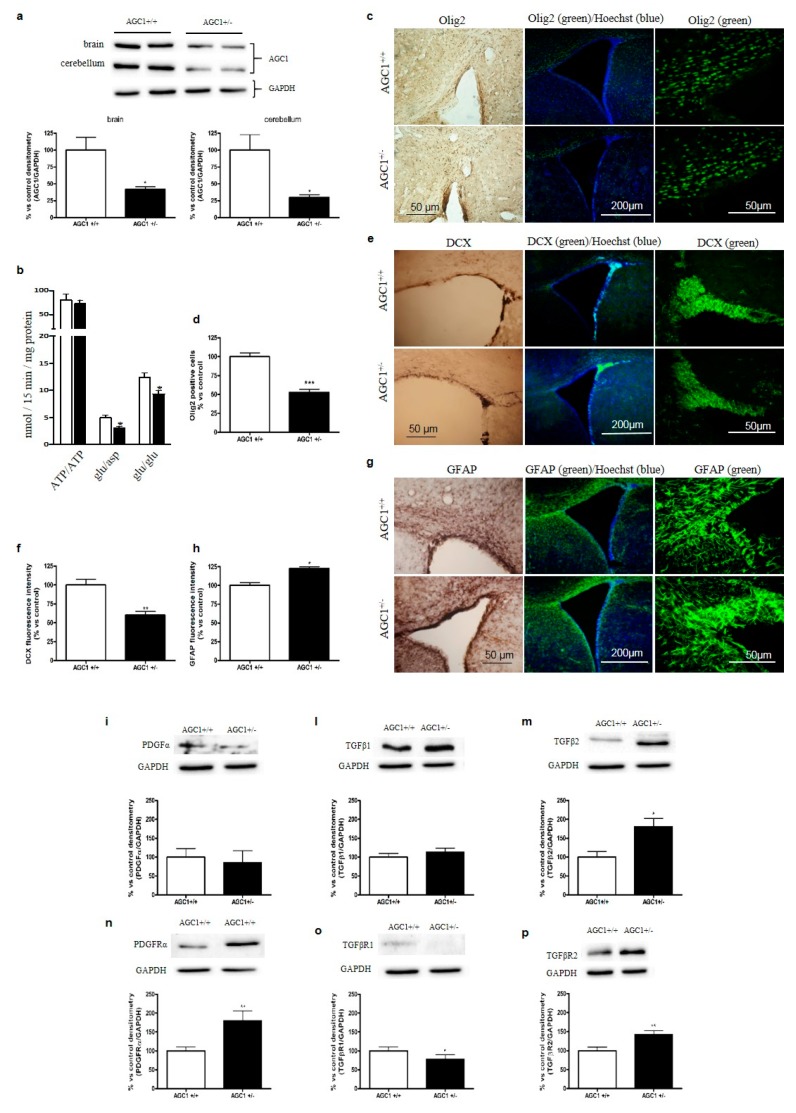
Proliferation deficits and dysregulation of PDGFα and TGFβ pathways in 21-day old AGC1^+/−^ mice. Western Blot analysis of AGC1 expression in 21-day old AGC1^+/+^ (*n* = 8) and AGC1^+/−^ (*n* = 8) mice in brain and cerebellum (**a**). GAPDH was used as reference loading control. Respective densitometric analyses are shown below. Bars represent the mean ± SE of 3 independent experiments performed in triplicate, * *p* < 0.05, compared to AGC1^+/+^ mice, *t*-test of Student. (**b**) Transport activities in brain mitochondria. [^14^C]ATP ext/ATP int (0.1 mM ext/20 mM ext), [^14^C]aspartate ext/glutamate int (0.05 mM ext/20 mM ext) and [^14^C]glutamate ext /glutamate int (0.1 mM ext/20 mM ext) were assayed in liposomes reconstituted with mitochondrial protein extracts isolated from AGC1^+/+^ (white column) and AGC1^+/−^ (black column) mouse brains. Transport activities were measured 30 min after the addition of the radiolabelled substrates. Data are the mean ± SD, *n* = 6, * *p* < 0.01 compared to liposomes reconstituted with AGC1^+/+^ mitochondrial extracts, one-way analysis with Bonferroni’s *post-hoc* test. Immunohistochemical and immunofluorescence analysis of Olig2^+^ cells (**c**), as well as doublecortin (DCX) (**e**) and Glial fibrillary acidic protein (GFAP) (**g**), respectively markers of OPCs, immature neurons and astrocytes, in the corpus callosum and subventricular zone of 21-day old AGC1^+/+^ and AGC1^+/−^ mice (scale bar = 300 μM). Cell count and fluorescence intensity analysis showed a significant reduction for Olig2^+^ (**d**) and DCX^+^ (**f**), while GFAP^+^ cells (**h**) were increased in AGC1^+/−^ mice. Bars are expressed as percentage vs. AGC1^+/+^ and represent the mean ± SE of three experiments for Olig2 and the mean ± SE of two experiments for DCX and GFAP. * *p* < 0.05, *** *p* < 0.001 compared to AGC1^+/+^ mice. Student’s t-Test. Western blot analysis and relative densitometries of PDGFα (**i**), TGFβ1 (**l**), TGFβ2 (**m**), PDGFαR (**n**), TGFβR1 (**o**) and TGFβR2 (**p**) expression in 21-day old AGC1^+/+^ and AGC1^+/−^ mice. Densitometry is the *ratio* between the expression level of each protein and of GAPDH as reference loading control and is expressed as percentage vs. AGC1^+/+^. Values are the mean ± SE of 3 independent experiments performed in triplicate, * *p* < 0.05, ** *p* < 0.01, compared to AGC1^+/+^, Student’s *t*-test.

In order to confirm in vivo the reduction of cell proliferation with concomitant induction of spontaneous differentiation and dysregulation of the PDGFα/TGFβs pathways observed in OPCs with impaired AGC1, we used an AGC1 deficiency mouse model. A murine model with inactivated AGC1 was generated in the C57BL/6N background with gene-trapping technology, by inserting a 6.5 kb VICTR 76 construct at intron 2–3 of the *SLC25A12* mouse gene (Texas A&M Institute for Genomic Medicine, see materials and methods for details). No live AGC1^−/−^ mice were detected among newborn mice, suggesting that the deletion of AGC1 activity may be lethal in mice with the C57BL/6N background. Therefore, we focused on AGC1^+/−^ mice and AGC1^+/+^ mice as controls, at 21 days after birth, since at this stage OPCs reach a peak in proliferation. Firstly, AGC1 levels were analysed by western blot in the brain and cerebellum of 21-day old mice; a significant reduction in AGC1 expression was observed in AGC1^+/−^ mice compared to controls (Figure 5a). Moreover, mitochondrial extracts from the brain of AGC1^+/−^ mice reconstituted in liposomes exhibited impaired AGC1 aspartate/glutamate and glutamate/glutamate exchange activities, when compared to liposomes reconstituted with mitochondrial extracts from the brain of control AGC1^+/+^ mice (Figure 5b).

Given that in the cell model of AGC1 deficiency we observed a reduction in OPC proliferation, we analysed whether also AGC1^+/−^ mice displayed OPC proliferation deficits when compared to AGC1^+/+^ mice. For this purpose, we performed immunohistochemical and immunofluorescence analyses of brain sections containing the SVZ, a region rich in neural stem cells (NSCs), which gives rise to neurons, astrocytes and OPCs. Interestingly, the SVZ showed a lower number of cells positive for p-H3, a widely used proliferation marker in the brain [21] (Supplemental Figure S4a), in the SVZ of AGC1^+/−^ mice when compared to AGC1^+/+^ mice, suggesting a lower proliferation of NSCs and/or other proliferating precursor cells. To further elucidate the nature of these proliferating cells, we performed immunofluorescence analysis in AGC1^+/+^ and AGC1^+/−^ mice for Olig2, a transcription factor highly expressed by OPCs [22]. A lower number of Olig2^+^ cells in AGC1^+/−^ mice was detected compared to AGC1^+/+^ mice, suggesting a reduced presence of OPCs (Figure 5c,d). In addition, immunofluorescence analysis for doublecortin (DCX), a microtubule-associated protein widely used as an immature neuronal specific marker linked to neurogenesis [23], revealed a lower intensity signal for DCX in AGC1^+/−^ mice compared to AGC1^+/+^ mice (Figure 5e,f). Furthermore, we detected more evident staining for the astrocytic marker GFAP (glial fibrillary acidic protein) in AGC1^+/−^ mice compared to AGC1^+/+^ mice (Figure 5g,h), suggesting an increased number of astrocytes. Overall, these data seem to confirm the reduction of OPC proliferation in our mouse model as observed in AGC1-silenced Oli-Neu cells and to show a lower number of OPCs and immature neurons with a parallel higher number of astrocytes, indicating a change in the proliferating neuron/glial cell ratio. To complete the analysis of oligodendrocyte proliferation and differentiation, we also measured the activity of CNPase and performed MBP immunohistochemistry, both known markers of mature oligodendrocytes [24,25]. As shown in Supplemental Figure S5b, these experiments showed no significant differences between AGC1^+/+^ and AGC1^+/−^ mice, thus suggesting that in our animal model the reduction in AGC1 expression and activity does not affect oligodendrocyte differentiation but rather OPC proliferation, in agreement with what observed in Oli-Neu cells.

Furthermore, we aimed to investigate whether the trophic factor alterations demonstrated in AGC1-silenced Oli-Neu cells could also be present in our in vivo model. We analysed the expression of PDGFα and TGFβ pathway members in whole brain tissue extracts from 21-day old AGC1^+/+^ and AGC1^+/−^ mice (Figure 5i–p). PDGFα receptor, mainly expressed by OPCs, was higher in 21-day old AGC1^+/−^ mice compared to control animals (Figure 5n); TGFβ2 levels were also higher in 21-day old AGC1^+/−^ mice compared to AGC1^+/+^ mice (Figure 5m), whereas TGFβ1 levels showed no differences (Figure 5l). TGFβ receptors 1 and 2 showed opposite expression patterns, being TGFβR1 less (Figure 5o) and TGFβR2 more expressed (Figure 5p), respectively, in AGC1^+/−^ mice compared to AGC1^+/+^ mice. All these data supported that a reduction in AGC1 expression leads to reduced OPC proliferation with a dysregulation in the PDGFα/TGFβ pathways; in vivo, a compensatory cascade may be triggered to attempt to sustain OPC proliferation in AGC1 ^+/−^ mice, although this is not enough.

### 2.6. OPC Proliferation and PDGFα/TGFβs Expression in Neurospheres From the SVZ of AGC1^+/+^ and AGC1^+/−^ Mice

**Figure 6:**
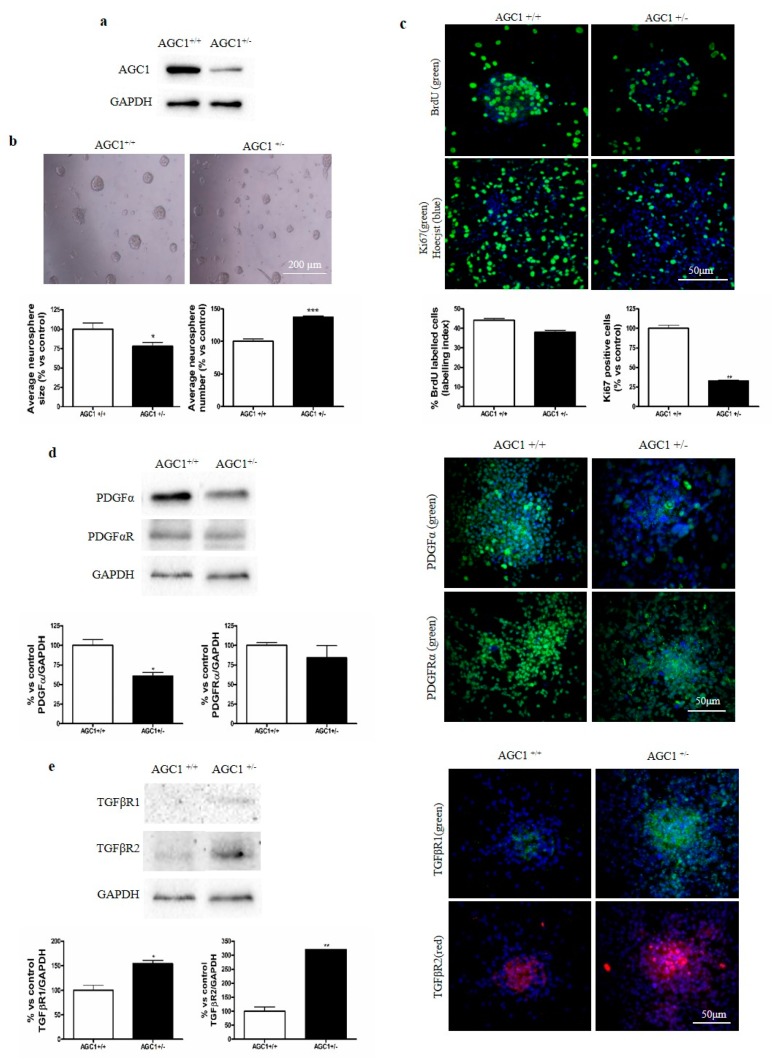
Altered proliferation and TGFβ receptor expression in neurospheres from the SVZ of AGC1^+/+^ and AGC1^+/−^ mice. Western Blot analysis confirmed reduced AGC1 expression in AGC1^+/−^ neurospheres compared to AGC1^+/+^ neurospheres (about 65–70% reduction). GAPDH was used as an endogenous control to normalize data (**a**). Bright field microscopy images (10X) of AGC1^+/+^ and AGC1^+/−^ neurospheres. AGC1^+/−^ neurospheres (right) showed heterogeneous morphology and appeared smaller than AGC1^+/+^ neurospheres. AGC1^+/−^ neurosphere cell number was also higher than AGC1^+/+^ neurospheres confirmed by newly formed AGC1^+/+^ and AGC1^+/−^ neurosphere size and number analysis after 4 days of incubation. * *p* < 0.05, *** *p* < 0.001 compared to AGC1^+/+^ neurospheres. Student’s *t*-test. Scale bar: 200 μm. (**b**). BrdU and Ki67 immunofluorescence 3D confocal microscopy images (40X) on AGC1^+/+^ and AGC1^+/−^ neurospheres, BrdU or Ki67 (green), nuclei (blue); BrdU and Ki67-positive cell count in AGC1^+/−^ neurospheres compared to AGC1^+/+^ neurospheres. ** *p* < 0.01 compared to AGC1^+/+^ neurospheres. Student’s *t*-test. Scale bar: 50 μm (**c**). Western blot analysis and relative densitometries of PDGFα and PDGFRα expression in AGC1^+/+^ and AGC1^+/−^ neurospheres. Densitometry is the ratio between the expression level of each protein and GAPDH as reference loading control and is expressed as percentage vs AGC1^+/+^ neurospheres. Immunofluorescence staining of PDGFα and PDGFRα in AGC1^+/+^ and AGC1^+/−^ neurospheres (nuclei were labelled with Hoechst). Scale bar: 50 μm. Values are the mean ± SE of 3 independent experiments performed in triplicate, * *p* < 0.05, compared to AGC1^+/+^ neurospheres, Student’s *t*-test (**d**). Western blot analysis and relative densitometries of TGFβR1 and TGFβR2 expression in AGC1^+/+^ and AGC1^+/−^ neurospheres. Densitometry is the ratio between the expression level of each protein and GAPDH as reference loading control and is expressed as percentage vs AGC1^+/+^ neurospheres. Immunofluorescence staining of TGFβR1 and TGFβR2 in AGC1^+/+^ and AGC1^+/−^ neurospheres (nuclei were labelled with Hoechst). Scale bar: 50 μm. Values are the mean ± SE of 3 independent experiments performed in triplicate, * *p* < 0.05, ** *p* < 0.01, compared to AGC1^+/+^ neurospheres, Student’s *t*-test (**e**).

**Figure 7:**
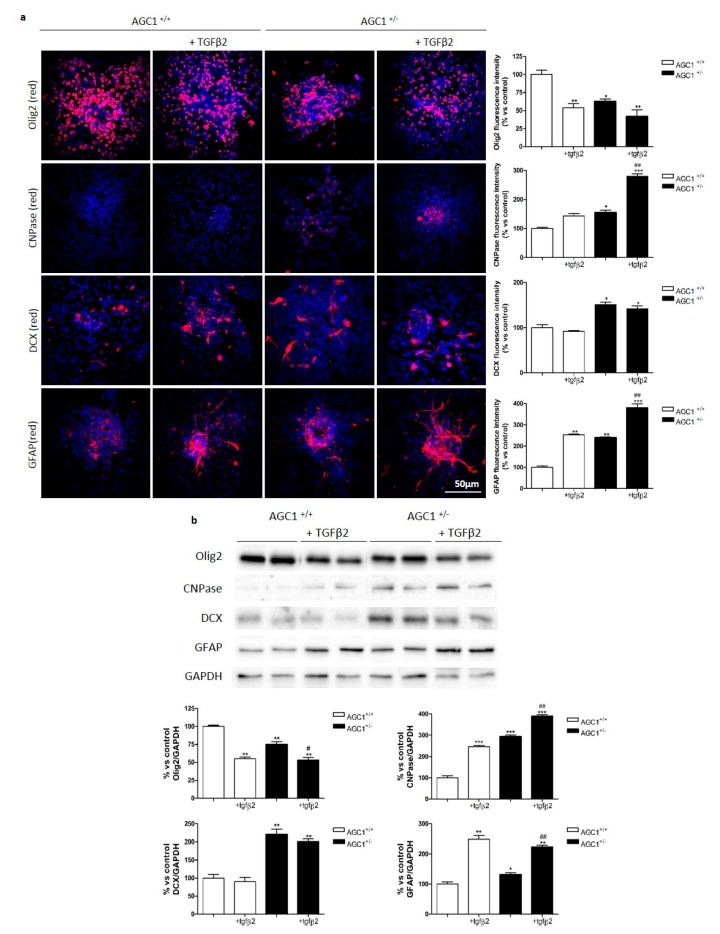
Effect of exogenous TGFβ2 on proliferation/differentiation in neurospheres from AGC1^+/+^ and AGC1^+/−^ mouse SVZ. Immunofluorescence staining in AGC1^+/+^ and AGC1^+/−^ neurospheres and following treatment with TGFβ2 after 4-day differentiation (**a**). Olig2, CNPase, DCX and GFAP used as specific markers for OPCs, mature oligodendrocytes, NSCs and astrocytes respectively (nuclei labelled with Hoechst). Scale bar: 50 μm. Analyses for Olig2^+^ and DCX^+^ cell number and CNPase^+^/GFAP^+^ fluorescence signal intensity evaluated with Fiji ImageJ2 software. Values are the mean ± SE of 3 independent experiments performed in triplicate, * *p* < 0.05, ** *p* < 0.01, *** *p* < 0.001 compared to control AGC1^+/+^ neurospheres, # *p* < 0.05, ## *p* < 0.01 compared to control AGC1^+/−^ neurospheres, Two-way ANOVA (Bonferroni’s post-test). Western blot analysis and relative densitometries of Olig2, CNPase, DCX and GFAP expression in AGC1^+/+^ and AGC1^+/−^ neurospheres and following treatment with TGFβ2 after 4-day differentiation (**b**). Densitometry is the ratio between the expression level of each protein and GAPDH as reference loading control and is expressed as percentage vs AGC1^+/+^ neurospheres. Values are the mean ± SE of 3 independent experiments performed in duplicate, * *p* < 0.05, ** *p* < 0.01, *** *p* < 0.001compared to AGC1^+/+^ neurospheres, # *p* < 0.05, ## *p* < 0.01 compared to control AGC1^+/−^ neurospheres, Two-way ANOVA (Bonferroni’s post-test).

To investigate more in detail the OPC proliferation deficit observed in AGC1 deficiency, neurospheres were derived from the SVZ of AGC1^+/+^ and AGC1^+/−^ mice. AGC1^+/−^ mouse neurospheres showed a lower expression of AGC1 (Figure 6a) and size, with parallel increased numbers (Figure 6b) compared to controls. By analysing Ki67 staining and BrdU incorporation (Figure 6c), neurospheres from AGC1^+/−^ mice showed a significantly lower level of proliferation compared to neurospheres from AGC1^+/+^ mice. Interestingly, AGC1^+/−^ neurospheres allowed to spontaneously differentiate for 7 days, showed a lower Olig2^+^ cell number with a parallel higher number of CNPase^+^, DCX^+^ and GFAP^+^ cells (Figure S5a) and expression of the same markers (Supplemental Figure S5b). Furthermore, as observed in Oli-Neu cells and in the in vivo model, western blot and immunofluorescence analysis demonstrated that AGC1^+/−^ mouse neurospheres showed a lower PDGFα expression (Figure 6d) and a significantly higher level of TGFβRs (Figure 6e) compared to AGC1^+/+^ neurospheres. These data indicate an OPC proliferation deficit with a parallel spontaneous induction of oligodendrocyte differentiation, probably related to dysregulation of the PDGFα/TGFβ pathways even in the neurosphere model, similar to what was observed in Oli-Neu cells. To further support the role of TGFβRs in AGC1^+/−^ OPC proliferation/differentiation dysregulation, neurospheres were treated with exogenous TGFβ2 for 4 days (Figure 7a,b). Immunofluorescence staining and western blot analysis show increased CNPase^+^ cell numbers in neurospheres from control animals indicating an induction of oligodendrocyte differentiation. This induction is significantly higher in AGC1^+/−^ neurospheres than in control neurospheres, where there is also a significant induction of GFAP^+^ cells, clearly indicating that higher levels of TGFβRs in AGC1^+/−^ neurospheres are involved in the dysregulation of OPC proliferation/differentiation.

## 3. Discussion

Mutations in the *SLC25A12* gene cause AGC1 deficiency, a rare infantile encephalopathy, which results in cerebral hypomyelination and low levels of N-acetyl aspartate (NAA) in the CNS due to the reduced activity of the mitochondrial carrier AGC1 [1,2]. While the effect of AGC1 impairment on biochemical pathways, such as MAS is well known [5,12,13,19], its direct role in CNS myelination remains largely unknown. By using both in vitro and in vivo models, here we showed for the first time that reduction in AGC1 expression and activity determines a deficit in OPC proliferation without reducing OPC differentiation into oligodendrocytes. This proliferation defect correlates with dysregulation in the expression of growth factors involved in OPC proliferation/differentiation, that is, PDGFα and TGFβs, as well as their receptors. Previous published data focused on neuronal cells revealed an important role of AGC1 in myelin synthesis [19]. NAA is generated in neurons by aspartate acetylation through aspartate-N-acetyltransferase and then transferred into oligodendrocytes where it provides acetyl groups for myelin lipid synthesis [1]. AGC1 down-regulation led to reduced growth and NAA production in undifferentiated Neuro2A mouse neuronal cell lines [19]. These data could provide information about the development of the main clinical phenotype observed in AGC1 deficiency patients, including cerebral atrophy and hypomyelination associated with reduced NAA levels [1,2]. Profilo and collaborators [19] hypothesized that in normally proliferating undifferentiated neuronal cells, AGC1 activity supports mitochondrial pyruvate oxidation leading to acetyl-CoA production in mitochondria, which is also necessary for lipid and NAA synthesis. Moreover, Ramos and colleagues observed hypomyelination in the cerebral cortex of AGC1^−/−^ mice, as well as a parallel increase in the number of immature oligodendrocytes, suggesting a defect in oligodendrocyte maturation. However, the same authors observed no defects in oligodendrocyte maturation in vitro [10]. On the other hand, down-regulation of AGC1 reduced MBP expression in primary OPCs, suggesting a direct role of AGC1 in the myelination process in oligodendrocytes [18]. Besides these studies, no other investigations on AGC1 impairment in OPCs have been performed. Given the importance of oligodendrocytes in myelin synthesis and their crosstalk with neurons, we hypothesized that the pathogenesis of AGC1 deficiency could involve an alteration of OPC proliferation/differentiation mechanisms. Following cell proliferation studies, we observed that siAGC1 Oli-Neu cells proliferated less than control cells, as also suggested by flow cytometry experiments. Furthermore, our data clearly demonstrated that the reduction of AGC1 expression does not impair db-cAMP-induced Oli-Neu cell differentiation. In addition, in the absence of db-cAMP, siAGC1 Oli-Neu cells displayed characteristics of premature cell differentiation, such as more elongated and branched morphology, a decrease in total filament number and an increase in average filament length and number of filaments per cell, as compared to control cells, suggesting that AGC1 impairment could induce Oli-Neu cell differentiation by itself.

Unlike previously reported data from AGC1-silenced Neuro2A cells [19], we did not observe significant alterations in mitochondrial function between control and siAGC1 Oli-Neu cells. In particular, AGC1 down-regulation did not seem to compromise glucose oxidation rate in Oli-Neu cells, perhaps due to compensation by AGC2 isoform expression, nor it changed mitochondrial morphology or membrane potential, suggesting that AGC1 down-regulation did not affect the mitochondrial network and activity. However, AGC1 silencing induced a slight yet significant increase of cytosolic ROS production, when compared to Oli-Neu control cells, whereas no difference in mitochondrial ROS production and in mitochondrial [Ca^2+^] was detected. Increased ROS production has been previously associated with enhanced differentiation in oligodendrocytes [26], which could partly explain the more differentiated morphology observed in siAGC1 Oli-Neu cells, although more likely other mechanisms besides increased ROS production may be involved in enhanced OPC differentiation In particular, we hypothesized that the observed proliferation deficit could be associated with an alteration of trophic factors essential for balancing oligodendrocyte proliferation and differentiation [27]. In fact, siAGC1 Oli-Neu cells showed a lower expression of PDGFα (involved in OPC proliferation) and a significant increase of TGFβ 1/2 (necessary for OPC differentiation), supporting the hypothesis that AGC1 reduced expression could lead to OPC proliferation impairment by favouring premature differentiation. While PDGFα receptor expression remained unchanged, TGFβ receptors 1 and 2 were expressed more in siAGC1 Oli-Neu cells, especially TGFβR2. This was unexpected, since previous reports showed that TGFβR2 deletion in oligodendrocyte progenitors caused hypomyelination and prevented development into mature myelinating oligodendrocytes [28]. It is conceivable that siAGC1 Oli-Neu cells express TGFβR2 because they might already be committed into a pre-differentiated state, yet their proliferation is still possible. To confirm this hypothesis, we observed that exposure of Oli-Neu cells to exogenous TGFβ2 reduced proliferation in both control and AGC1-silenced cells but the induction of differentiation was much more evident in silenced Oli-Neu, thus indicating that the increased level of TGFβ receptors could make these cells more prone to differentiation. It is noteworthy that the decrease in AGC1 expression and activity could affect gene expression in OPCs. Through high throughput proteomic approaches, it was observed that AGC1 may interact [29] with transcription factors such as c-myc, which is known to play a crucial role in regulating OPC proliferation and differentiation [28].

Interestingly, it has been demonstrated that inflammation could affect myelination by acting on OPCs during development [30]. These data are in agreement with our observation and further suggest that not only mature oligodendrocytes but also OPC defects could play a role in demyelinating diseases. Furthermore, it has been demonstrated that a reduction in OPC generation due to their precocious differentiation into oligodendrocytes can impair functional remyelination in response to a demyelinating insult, at least in the *corpus callosum* (CC) [31]. We validated our in vitro results in *SLC25A12* heterozygous knockout mice (AGC1^+/−^ C57BL6/N background). AGC1^+/−^ mice display reduced AGC1 carrier expression (about 60%) compared to AGC1^+/+^ mice. AGC1 knockout mice (AGC1^−/−^) have previously been described in the literature as an AGC1-deficiency model. The mouse model developed by Jalil and collaborators [9] and used by Satrústegui and collaborators was generated in the SVJ129/C57BL background with an insertion at intron 13, leading to an early stop in the AGC1 protein. These AGC1^−/−^ mice showed growth retardation, epileptic seizures and reduced myelination (typical AGC1 deficiency symptoms), as well as global cerebral atrophy, alteration of neurofilament distribution in cortical neurons and Purkinje cell abnormalities in the cerebellum, yet they all died at weaning [9,10,11]. In our case no AGC1^−/−^ mice were born alive, this could be explained by the fact we generated a mouse model with a different genetic background by using an insertion at intron 2-3, thus completely blocking AGC1 expression. Since currently identified AGC1 mutations determine a reduction of activity compared to wild-type AGC1 and not the total absence of mitochondrial carrier activity [2], AGC1^+/−^ mice could be considered a good model of the disease. AGC1^+/−^ mice did not show any suffering phenotype or apparent changes that compromise normal behavioural functions. Furthermore, although AGC1^+/−^ mice showed a smaller SVZ and overall brain ventricle size (data not shown), they were not distinguishable from AGC1^+/+^ mice. We performed studies on 21-day old AGC1^+/+^ and AGC1^+/−^ mice since this stage represents the peak of OPC proliferation. Significant reduction of proliferating cells (positive for proliferation markers, such as phosphorylated histone H3 on serine 10 and Ki67) in the SVZ and the *corpus callosum*, as well as a reduction of Olig2 (OPC marker) and DCX (immature neuron marker) positive cell number were observed in AGC1^+/−^ mice. Since DCX is known to be essential for neuronal migration, cell proliferation during neurogenesis and for the development of a functional brain [32], reduced DCX signal could explain the differences in SVZ and brain ventricle size observed in AGC1^+/−^ mice compared to AGC1^+/+^ mice. Our data suggest that proliferation defects actually affected OPCs in AGC1^+/−^ mice, in agreement with our in vitro results. Moreover, reduced AGC1 expression had no macroscopic effects on mature oligodendrocytes and myelination, since no significant differences in MBP^+^ and CNPase^+^ cells were observed through immunohistochemical and biochemical analysis. To further support the hypothesis that reduced AGC1 expression affect OPC proliferation without altering their differentiation into oligodendrocytes, neurospheres derived from the SVZ of AGC1^+/+^ and AGC1^+/−^ mice were used. Neurospheres are considered a solid tool to study neurogenesis and gliogenesis, giving a representation of the number and type of stem cells in a particular niche. In particular, adhesion to the substrate leads to differentiation into neurons, astrocytes and oligodendroglia [33,34]. Interestingly, neurospheres from AGC1^+/−^ mice displayed reduced cell proliferation and, when allowed to spontaneously differentiate, reduced Olig2^+^ cell number with a parallel increase in CNPase^+^, DCX^+^ and GFAP^+^ cells. As in our in vitro model, an imbalance in PDGFα and TGFβ pathways was also observed in our in vivo mouse model: while PDGFα expression did not change in 21-day old mice, TGFβ2 expression was increased, confirming that OPCs proliferated less because they may differentiate prematurely. PDGFα receptor was more expressed in 21-day old AGC^+/−^ mice compared to AGC1^+/+^ mice, in agreement with literature data, showing that PDGFRα expression is a key event in OPC ontogenesis [35,36,37], which could be an attempt of AGC1^+/−^ OPCs to maintain their proliferative status. On the other hand, TGFβR2 was more expressed in 21-day old AGC1^+/−^ mice as observed in AGC1 silenced Oli-Neu cells indicating that OPCs differentiated earlier into mature oligodendrocytes [28]. While TGFβR1 was less expressed in AGC1^+/−^ mice, the increased expression of TGFBR2 may be able to promote oligodendrocyte differentiation, considering also that TGFBR2 is able to autophosphorylate and then form a receptor complex with TGFβR1 in order to trigger signal transduction [37]. More interestingly, neurospheres from AGC1^+/−^ mice showed reduced PDGFα with parallel increased TGFβR expression and, as expected, exogenous TGFβ treatment determines an induction in oligodendrocyte differentiation, significantly higher than in AGC1^+/+^ mice. All these results confirmed our in vitro data, supporting that alterations induced by AGC1 reduced activity could impair the physiological crosstalk between neurons and OPCs through growth factors, necessary for OPC proliferation and neuronal survival. These data not only agree with previous data published by Ramos and collaborators [10], who observed no alteration in oligodendrocyte maturation both in vitro and in vivo *but* could also contribute to explain their results. Furthermore, our data support the observation that in demyelinating diseases, including multiple sclerosis, the ability to regenerate oligodendrocytes depends on OPC availability [17]. The treatment of a patient affected by AGC1 deficiency with a ketogenic diet improved disease phenotype [38]. A ketogenic diet is often used in the treatment of epilepsy and it has positive effects against neurodegeneration [39]. Although, the biochemical, molecular and cellular mechanisms underlying this positive nutritional approach to favour myelination have not been completely clarified [40], we hypothesized that the reduced glucose oxidation in neurons with AGC1 deficiency in the presence of an alternative source of sufficient acetyl groups from ketones could compensate the metabolic defect and promote myelination [19]. Whether a ketogenic diet might also influence oligodendrocyte proliferation and/or maturation in patients with AGC1 deficiency will deserve further investigation.

## 4. Materials and Methods

### 4.1. Cell Cultures

Oli-Neu cells (kindly provided by Dr. Jacky Trotter, University of Mainz, Germany, RRID:CVCL_IZ82) were grown on poly-l-lysine (10 μg/mL; Sigma-Aldrich, St Louis, MO, USA) coated Petri dishes in SATO medium (Dulbecco’s Modified Eagle’s medium DMEM medium, 2 mM glutamine, 10 μg/mL insulin, 5.5 μg/mL transferrin, 38.72 nM sodium selenite, 100 µM putrescine, 520 nM l-thyroxine (T4), 500 nM triiodo-l-thyronine (T3), 200 nM progesterone, 25 μg/mL of gentamycin) supplemented with 1% heat-inactivated horse serum (HS) in a humidified, 5% CO_2_ incubator at 37 °C. Cell culture medium and all chemicals were from Sigma-Aldrich, except for insulin-transferrin-sodium selenite 100X supplement (Life Technologies Corporation, Grand Island, NY, USA). When cells reached confluence, they were washed once with Phosphate Buffer Solution (PBS) and detached with 0.01% trypsin-0.02% EDTA- Hank’s Balanced Salt Solution (HBSS, Sigma-Aldrich). To induce differentiation, 1mM (N6,2′-o-dibutyryl)-adenosine-3′,5′-mono-phosphate (db-cAMP, Sigma-Aldrich) was added every day for 3DIV.

### 4.2. Stable Clone Generation

Oli-Neu stable cell clones were prepared as follows: 3 × 10^5^ cells were plated on 60-mm diameter poly-l-lysine (10 µg/mL) coated Petri dishes (Corning). After 24 h of incubation, cells were transfected by using Lipofectamine 2000 reagent (Thermo Fisher Scientific, Waltham, MA, USA) following the manufacturer’s instructions. Briefly, 5 µg of pLKO.1-puro DNA plasmid (Sigma-Aldrich) containing a mismatch control (scrambled) sequence:

5′-CCGGTACAACCAACGCACGCTAATCTCGAGATTAGCGTGCGTTGGTTGTTTTTTG-3′

or an AGC1 silencing sequence:

5′-CCGGTGCTTGCAGACCTATATAATGCctcgagGCATTATATAGGTCTGCAAGCTTTTT-3′)

Digested with AgeI/EcoRI and cloned into the pLKO.1-puro vector, as previously published [41] and 10 µL of Lipofectamine 2000 reagent were diluted each in 250 µL of Opti-MEM medium (Thermo Fisher Scientific, Waltham, MA, USA). Both solutions were then gently mixed together. After 5 min of incubation, the plasmid DNA-lipofectamine solution was added directly into each Petri dish respectively. Twenty-four hours after transfection, the cell medium was replaced with fresh medium containing 1 µg/mL puromycin (Sigma-Aldrich), in order to select stably transfected cells. The cell medium was replaced every day. When puromycin resistant cells reached confluence, they were trypsinized and grown in SATO medium supplemented with 1% HS and 1 µg/mL puromycin on 60-mm diameter poly-l-lysine (10 µg/mL) coated Petri dishes.

### 4.3. TGFβ2 Treatment

Oli-Neu cells were plated at the density of 1 × 10^5^ in 6-well plates containing poly-l-lysine (10 µg/mL) coated glass coverslips. Cells were allowed to adhere for 2 h before adding 10 ng/mL TGFβ2 (final concentration; Sigma-Aldrich). After 24 h, cells were fixed for 30 min at RT with 4% PFA in phosphate buffer (0.194 M Na_2_HPO_4_, 0.026 M NaH_2_PO_4_) and stored in PBS at 4°C until used for cell filament measurement and immunofluorescence or resuspended in cell lysis buffer (50 mM Tris pH 7.4, 1 mM EDTA, 1% SDS, 10 µL/mL protease and phosphatase inhibitor cocktails) for western blot analysis.

### 4.4. Cell Count

1 × 10^5^ Oli-Neu cells/well were plated in duplicate (3 different experiments) in 6-well plates. After 24 h, cells were detached with a solution containing 0.01% trypsin-0.02% EDTA-HBSS (Sigma-Aldrich). Cells were then diluted with an equal amount of DMEM-10% HS and centrifuged for 5 min at 400× *g*. The cell pellet was resuspended in 1 mL of SATO medium and cell number was determined using a Neubauer chamber.

### 4.5. Cell Filament Number and Length Measurement

1 × 10^5^ Oli-Neu cells were plated in 35-mm Petri dish. After 24 h, images from 5 randomly selected fields for each Petri dish were acquired with an Eclipse TS100 (Nikon, Shinjuku, Japan) microscope using a 10X objective. Filament length was measured with Fiji ImageJ2 software (Fiji, RRID:SCR_002285, developed by the National Institutes of Health, NIH, USA) by using the reference scale bar and the SET SCALE function (Analyze menu) in order to set the scale bar distance in pixels and the actual distance in micrometres. After tracing each cell filament with the segmented line function, the MEASURE function (Analyze menu) was used to determine filament length in micrometres. Filament number was determined directly from individual filament length measurements.

### 4.6. Oli-Neu Bromodeoxyuridine (BrdU) Counting Following Immunofluorescence and Elisa Incorporation Assay

For Oli-Neu BrdU immunofluorescence, 1 × 10^5^ cells/well were plated in 6-well plates containing poly-l-lysine (10 µg/mL) coated glass coverslips. Cells were allowed to adhere for 4 h before adding 10 μM BrdU (final concentration). After 24 h, cells were fixed for 30 min at room temperature (RT) with 4% paraformaldehyde (PFA) in phosphate buffer (0.194 M Na_2_HPO_4_, 0.026 M NaH_2_PO_4_) and stored in PBS at 4 °C until used. Coverslips were incubated with 2 N HCl for 30 min at RT, washed 4 × 10 min with PBS and then 3 × 5 min in PBS-0.1% Triton (PBS-T). Aspecific sites were blocked by incubating at RT for 1 h in blocking buffer (PBS-T + 5% goat serum). Coverslips were incubated overnight at 4 °C in a humidified chamber with a rat anti-BrdU monoclonal antibody (Abcam Cat# ab6326, RRID:AB_305426, Lot# GR251710-3, 1:500 dilution in PBS-T + 2% goat serum). The next day, coverslips were washed 3 × 10 min with PBS-T, incubated for 2 h with goat anti-rat Alexa 488-conjugated antibody (Abcam Cat# ab150157, RRID:AB_2722511, Lot# GR290139-4, 1:1000 in PBS-T + 2% goat serum) and washed 3 × 10 min with PBS-T, 5 min with PBS and then incubated for 5 min with Hoechst 33258 (2 µg/mL, Sigma-Aldrich). Coverslips were then washed for 5 min in PBS and then mounted on glass slides with UltraCruz Mounting medium (Santa Cruz, Dallas, TX, USA), sealed with nail polish and stored at 4 °C until used. Total nuclei stained with Hoechst and BrdU positive nuclei were counted in 5 randomly selected fields for each coverslip. Labelling index was expressed as the *ratio* of BrdU positive/Hoechst stained cells.

For BrdU ELISA incorporation assay, 1 × 10^4^ Oli-Neu cells/well were plated on 96-well plates. Cells were allowed to adhere for 4 h and 10 μM BrdU (final concentration) was added to each well. BrdU was incubated for 6 and 24 h respectively. BrdU incorporation was assayed using a BrdU cell proliferation kit (Roche, Risch-Rotkreuz, Switzerland), following the manufacturer’s instructions. Absorbance was read at 490 nm with a reference wavelength of 405 nm.

### 4.7. Lactic Acid Measurements

Lactic acid release by Oli-Neu cells was quantified in cell conditioned media harvested after the indicated incubation times by an enzymatic assay in spectrophotometry, as previously described [41].

### 4.8. Aequorin and Luciferase Luminescence Measurement

Oli-Neu cells were transiently transfected with plasmids carrying the coding sequence of recombinant aequorins selectively targeted to the cytosol (cytAEQ) or mitochondria (mtAEQmut) and of recombinant luciferase targeted to mitochondria (mtLuc) [42]. Transfected cells were incubated for 1 h at 37 °C with Krebs–Ringer modified buffer (KRB; 125 mM NaCl, 5 mM KCl, 1 mM Na_3_PO_4_, 1 mM MgSO_4_, 5.5 mM glucose and 20 mM 4-(2-hydroxyethyl)-1-piperazineethanesulfonic acid [HEPES], pH 7.4, at 37 °C) supplemented with 1 mM CaCl_2_ and 1 g/L glucose (+ 5 µM coelenterazine for aequorin reconstitution). Cells were subsequently perfused in the same buffer (+ 20 µM luciferin for luciferase assays) in a purpose-built luminometer where they were stimulated with 500 μM Carbachol (Cch). Aequorin experiments were terminated by lysing the cells in a hypotonic solution with 0.1 mM digitonin and 10 mM CaCl_2_ and light output was collected and calibrated in [Ca^2+^], as previously described [43]. In luciferase assays, data were expressed as mtLuc light output of cells.

### 4.9. Cell Fluorescence Analysis

Measurement of intracellular reactive oxygen species was performed by loading cells with 5 µM 5-(and-6)-chloromethyl-2′,7′-dichlorodihydrofluorescein diacetate, acetyl ester (CM-H2DCFDA; Thermo Fisher Scientific, Waltham, MA, USA) for 20 min at 37 °C and green fluorescence was analysed with a Tali^®^ Image-Based Cytometer. Mitochondrial hydrogen peroxide levels were measured in Oli-Neu cells cultured on 24 mm glass coverslips and transfected with the ratiometric fluorescent probe with mitochondrial localization pHyPer-dMito (mt-HyPer) [20]. After 24 h of expression, cells were maintained in KRB supplemented with 1 mM CaCl_2_ and 1 g/L glucose and placed in an open Leyden chamber on a 37 °C thermostated stage. 494/406 nm excitation filters and a 500-nm long-pass beam splitter were used and an image pair was obtained every 200 ms with a 40X objective. For a ratiometric measurement, at the end of each measurement, the efficiency of the probe was ascertained by adding H_2_O_2_ as reference. Fluorescence data were expressed as emission ratios. The experiments were performed on a Cell^R Olympus multiple wavelength high-resolution epi-fluorescence microscope. Mitochondrial inner membrane potential (Ψm) and mitochondrial morphology were measured by loading the cells with 20 nM tetramethyl rhodamine methyl ester (TMRM; Thermo Fisher Scientific, Waltham, MA, USA) for 30 min at 37 °C. Images were taken on an inverted Nikon LiveScan Swept Field Confocal Microscope (*SFC*) Eclipse Ti equipped with NIS-Elements microscope imaging software (Nikon Instruments, S.p.A Campi Bisenzio Firenze, Italy, RRID:SCR_014329). TMRM fluorescence intensities (exc. 560 nm; emis. 590–650 nm) were imaged every 5 s with a fixed 20 milliseconds exposure time. At the end of the experiments, 10 μM Carbonyl cyanide-4-(trifluoromethoxy)phenylhydrazone (FCCP) was added after 240 acquisitions to completely collapse the Ψm and subtract non-mitochondrial TMRM fluorescence, as previously described [44]. For mitochondrial morphology experiments, 51-plane z-stacks where acquired with a voxel dimension of 133 nm × 133 nm × 200 nm (X × Y × Z). The mitochondrial network, described in number and volume and 3D renders were obtained with Imaris 4.0 (Bitplane, Zurich, Switzerland).

### 4.10. Brain Mitochondria Isolation and Measurement of AGC1 Activity

In order to isolate mitochondria from mouse brain, a modified protocol from Grove and Bruckey was used [45]. Fresh brains from AGC1^+/+^ and AGC1^+/−^ mice were homogenized with a Potter homogenizer at 1000× *rpm*/30 strokes in a buffer containing 10 mM Hepes, 200 mM mannitol, 70 mM sucrose, 1 mM EDTA pH 7.6, 10 µL/mL protease and phosphatase inhibitor cocktails. Nuclei were pelleted by centrifugation at 800× *g* for 10 min at 4 °C. The cytoplasmic supernatant fraction (CF) was then centrifuged at 14000× *g* for 20 min. The crude mitochondrial pellet was resuspended in 500 µL of isotonic buffer (10 mM Hepes, 200 mM mannitol, 70 mM sucrose, 1 mM EDTA pH 7.6, 10 µL/mL protease and phosphatase inhibitor cocktails), centrifuged at 14,000× *g* for 20 min and then stored at −80 °C until used. Total protein sample content was determined by using the Lowry quantification method [46].

For AGC1 activity measurements, mitochondria were solubilized (0.6 mg protein/mL) in a buffer containing 3% Triton X-114, 10 mM Pipes pH 6.5, 1 mM EDTA and 4 mg/mL cardiolipin After 1 h of incubation on ice, extracts were centrifuged (12500× *g*, 10 min at 4 °C) to remove membranes and other unsolubilized impurities. 30 µg of solubilized mitochondria were then reconstituted in liposomes in the presence of 1% Triton X-114, 10 mM Pipes pH 6.5 and 10 mM l-glutamate or ATP through an Amberlite column XAD-2 (Sigma-Aldrich) (4.0 × 0.5 cm), as previously described [47]. All operations were carried out at 4 °C, except for the passage through the Amberlite column conducted at RT. Subsequently, the external substrate was removed from proteoliposomes by gel filtration chromatography on Sephadex G-75 columns (15 cm × 0.7 cm) pre-equilibrated with a buffer containing 10 mM PIPES pH 6.5 and 50 mM NaCl. Transport activity was determined by measuring the incoming flow of radiolabelled substrate into proteoliposomes. More in detail, transport was started at 25 °C by adding l-[^14^C]aspartate, l-[^14^C]glutamate or [^14^C]ATP to reconstituted proteoliposomes, at the indicated concentrations and terminated after 15 min by adding 8 mM pyridoxal5′-phosphate and 6 mM bathophenanthroline according to the “inhibitor-stop” method [5]. In control samples, inhibitors were added at time zero together with the labelled substrate. Lastly, the external substrate was removed by gel filtration on Sephadex G-75 column (8 × 0.6 cm) and the radioactivity incorporated in the eluted proteoliposomes was measured in a liquid phase scintillator (LS 6000 IC Beckman Coulter S.r.L. Milano, Italy). Experimental values were corrected by subtracting control values and transport activity was calculated as previously described [47].

### 4.11. AGC1^+/−^ Mice Experiments

C57BL/6N AGC1^+/−^ mice were generated with gene-trapping technology by the Texas A&M Institute for Genomic Medicine (Houston, TX, USA). Briefly, the 6.5 kb VICTR 76 targeting construct was designed to insert at intron 2-3 of the *SLC25A12* mouse gene. The linearized targeting vector was electroporated into C57BL/6N-derived ES cells. Correctly targeted ES cell clones were identified and used to develop mice, which were mated with C57BL/6N females. Mice used for experiments were obtained from departmental animal house, either from AGC1^+/−^ x AGC1^+/−^ matings or AGC1^+/−^ x AGC1^+/+^ matings, always using AGC1^+/+^ littermates as controls and were kept in controlled conditions of temperature and humidity with a light/dark cycle of 12 h, under veterinary surveillance for animal health and comfort. Animals were fed *ad libitum* with the Teklad global diet 2018 (Envigo, USA). All animal experiments were authorized by a local bioethical committee (Protocol n° 3/79/2014) and performed in agreement with the Italian and European Community law (Directive 2010/63/EU) on the use of animals for experimental purposes and adherence to the ARRIVE Reporting Guidelines.

Genotyping was performed at 14 days of age by extracting DNA from mouse tail tips by using Extract-N-Amp tissue extraction kit (Sigma-Aldrich) following the manufacturer’s instructions and performing a PCR assay (36 cycles). The following set of PCR primers were used in order to identify AGC1^+/+^, AGC1^+/−^ and AGC1^−/−^ mice.

IST11936G6-Forward. 5′-GGAGACTGACAGTCAACAAG-3′ (all animals). Tm = 52.76 °C

IST11936G6-Reverse. 5′-GGCATTTGCACACCGTGGA-3′ (AGC1^+/+^ and AGC1^+/−^ animals). Tm = 58.26 °C

Downstream Reverse. 5′-CCAATAAACCCTCTTGCAGTTGC-3′ (AGC1^+/−^ and AGC1^−/−^ animals). Tm = 58.30 °C

Two separate PCR reactions per sample were performed and amplification products IST11963G6-Forward + Downstream Reverse (342 bp), expected in AGC1^+/−^ and AGC1^−/−^ animals; IST11963G6-Forward + IST11963G6-Reverse (369 bp) expected in AGC1^+/+^ and AGC1^+/−^ animals reactions were resolved on a 2% agarose/TBE gel. Thus, for AGC1^+/+^ animals one 369 bp band was expected, for AGC1^+/−^ animals both a 369 and 342 bp band were expected, while for AGC1^−/−^ animals only one 342 bp band was expected.

At 21 days after birth, animals were sacrificed by decapitation, brains were then collected and stored at −80 °C until needed for western blot analysis (*n* = 21). For tissue immunohistochemistry, 21-day old AGC1^+/+^ and AGC1^+/−^ mice were anesthetized with 2% xilor and 100 mg/kg zoletil, then transcardially perfused with PBS and with 4% PFA in phosphate buffer (0.194 M Na_2_HPO_4_, 0.026 M NaH_2_PO_4_) (*n* = 16). Brains were then promptly removed and stored overnight in 4% PFA/phosphate buffer at 4 °C. The next day, fixed brains were stored overnight in 18% sucrose/phosphate buffer and then stored at −80 °C until needed.

### 4.12. Neurosphere Generation

Subventricular zone (SVZ) microdissection was performed in adult male mice (8-month old; 3 AGC1^+/+^ and 3 AGC1^+/−^ mice respectively) [34]. Dissected tissues were mechanically disaggregated in Hank’s Balanced Salt Solution (HBSS: HEPES 3.9 mg/mL, NaHCO_3_ 0.5 mg/mL, Glucose 0.9 mg/mL, penicillin/streptomycin 0.5%) and centrifuged at 300× *g* for 5 min. The pellet was resuspended in papain solution (0.2 mg/mL EDTA + 0.66 mg/mL Papain + 0.2 mg/mL cysteine in HBSS) and placed in a centrifuge tube at 37 °C for 20 min in a water bath, shaking every 5 min. The pellet was then resuspended in HBSS and left in a water bath at 37 °C for another 10 min. Papain was inhibited by the addition of DMEM-F12 (Gibco Life Technologies, Waltham, MA, USA) supplemented with insulin from bovine pancreas (Sigma-Aldrich) (10 µg/mL) and the sample was centrifuged at 300× *g* for 5 min. The pellet was resuspended in HBSS and centrifuged at 400× *g* for 5 min, the supernatant was discarded and the pellet was resuspended in DMEM-F12 (Gibco) supplemented with 2 mM glutamine, insulin (10 µg/mL), 20 ng/mL Epidermal Growth Factor (EGF; PeproTech EC, London, UK) 20 ng/mL Fibroblast Growth Factor-2, (FGF2; PeproTech), 1% N2 (Thermo Fisher Scientific, Waltham, MA, USA), 1% B27 (Thermo Fisher) and 10 units/mL penicillin and 10 µg streptomycin.

(Sigma-Aldrich). In order to induce neurosphere growth, EGF and FGF growth factors were added every other day (FGF concentration was halved by the third passage onwards (10 ng/µL); neurospheres were passaged every week (7 days of growth) by mechanical procedures. For this purpose, neurospheres were collected in DMEM-F12 medium, centrifuged at 300× *g* for 5 min and the pellet was resuspended in sterile PBS (4–5 times). Neurospheres were then centrifuged at 300× *g* for 5 min, the supernatant was removed and 1 mL of Acutase (Aurogene Srl, Roma, Italy) was added to the pellet by gently resuspending 2 times and incubated at 37 °C for 5 min. 4 mL of DMEM F12 were then added to the neurosphere suspension and then centrifuged at 300× *g* for 5 min. Single cells were counted and plated in complete DMEM F12 supplemented with insulin (10 µg/mL), 1% N2, 1% B27, 20 ng/mL EGF, 20 ng/mL FGF, 2 mM glutamine and 10 units/mL penicillin and 10 ug/mL streptomycin (Sigma-Aldrich).

### 4.13. Neurosphere Proliferation and BrdU Assay

To evaluate the proliferation and growth rate of SVZ-derived NSCs from AGC1^+/+^ and AGC1^+/−^ mice, neurospheres were plated as single stem cells in 96 multiwell plates (5 × 10^3^ cells/well) in complete DMEM-F12 medium after passage 3 and allowed to grow at 37 ˚C and 5% CO_2_ in an incubator. After 4 days in culture, 5 different image fields per well were acquired by using an eclipse TE 2000-s microscope (Nikon) in bright field mode using a 10X objective. Acquired images were then analysed with Fiji ImageJ2 software using the publicly available cell colony edge macro [48] and only neurospheres with an area bigger than 400 μm^2^ were considered; results were expressed as average neurosphere number and average neurosphere size.

To evaluate BrdU incorporation, AGC1^+/+^ and AGC1^+/−^ mouse neurospheres were passaged as single cells (1.43 × 10^5^ single cells/6-cm diameter Petri dish) in complete DMEM-F12 medium supplemented with 10 μM BrdU (Sigma-Aldrich). BrdU was added every day for 4 days and then 30 neurospheres were plated on 13-mm coverslips previously treated with poly-l-lysine (10 µg/mL) and then fibronectin (1 µg/mL, 37 °C incubation for at least 3 h) in complete DMEM-F12 medium supplemented with BrdU. After 24 h, neurospheres were fixed with 4% PFA for 30 min, washed with PBS and stored at 4 °C in PBS until used. Experiments were conducted in duplicate with 3 replicates per condition.

### 4.14. Neurosphere Treatment with Exogenous TGFβ

AGC1^+/+^ and AGC1^+/−^ SVZ-derived neurospheres were counted and plated on 13-mm coverslips (50 neurospheres/well) previously treated with poly-l-lysine (10 µg/mL) and then fibronectin (1 µg in complete DMEM-F12 medium with 10 ng/mL TGFβ2 (Sigma-Aldrich) for 4 days in an incubator at 37 °C. After 4 days, neurospheres were fixed with 4% PFA for 30 min, washed with PBS and stored at 4 °C in PBS until used for immunofluorescence analysis or resuspended in cell lysis buffer (50 mM Tris pH 7.4, 1 mM EDTA, 1% SDS, 10 µL/mL protease and phosphatase inhibitor cocktails) for western blot analysis.

### 4.15. Cell and Tissue Sample Preparation and Western Blot Analysis

Whole cell and neurosphere lysate samples were obtained by washing cell cultures once with PBS and resuspending cell cultures in cell lysis buffer (50 mM Tris pH 7.4, 1 mM EDTA, 1% SDS, 10 µL/mL protease and phosphatase inhibitor cocktails). Mouse tissue samples were lysed in tissue lysis buffer (10 mM Hepes, 200 mM mannitol, 70 mM sucrose, 1% NP40, 1 mM EDTA pH 7.6, 10 µL/mL protease and phosphatase inhibitor cocktails) with a Potter homogenizer at 1000 rpm/30 strokes. All samples were sonicated with a Branson 250 digital sonifier for 3 pulses of 2 s each (waiting for 5 s between each pulse) at 10% power output and stored at −80 °C until used. Total protein sample content was determined by using the Lowry quantification method [45] and 30 µg of each sample with Laemli loading buffer (1M Tris-HCl pH 6.8; 20% sodium dodecyl sulphate; 0.4 µL/mL glycerol; 2 g/L bromophenol blue and 2M dithiothreitol; all from Sigma-Aldrich), were loaded per lane for western blot analysis.

Samples were resolved in SDS-PAGE (Sodium Dodecyl Sulphate–Polyacrylamide Gel Electrophoresis), before electroblotting. Membranes were incubated overnight with primary antibodies against AGC1 (Santa Cruz Biotechnology Cat# sc-271056, RRID:AB_10608837, Lot# L1312), PDGFα (Santa Cruz Biotechnology Cat# sc-7958, RRID:AB_2161914, Lot# C0204), PDGFRα (Santa Cruz Biotechnology Cat# sc-338, RRID:AB_631064, Lot# F1614), TGFβ1 (Santa Cruz Biotechnology Cat# sc-146, RRID:AB_632486, Lot# H2907), TGFβ2 (Santa Cruz Biotechnology Cat# sc-90, RRID:AB_2303237), Lot# G0607), TGFβ3 (Santa Cruz Biotechnology Cat# sc-82, RRID:AB_2202303, Lot# L2308), TGFβR1 (Santa Cruz Biotechnology Cat# sc-398, RRID:AB_632493, Lot# G0314), TGFβR2 (Santa Cruz Biotechnology Cat# sc-17792, RRID:AB_628349, Lot# D1813), CNPase (Cell Signalling Technology Cat# 5664P, RRID:AB_10705455, Lot# 2) and Glyceraldehyde 3-phosphate dehydrogenase GAPDH (Santa Cruz Biotechnology Cat# sc-32233, RRID:AB_627679, Lot# B0514) and then with HRP-linked secondary antibodies goat anti-rabbit (Santa Cruz Biotechnology Cat# sc-2004, RRID:AB_631746, Lot# D1216), goat anti-mouse (Santa Cruz Biotechnology Cat# sc-2005, RRID:AB_631736, Lot# B1616), donkey anti-goat (Santa Cruz Biotechnology Cat# sc-2020, RRID:AB_631728, Lot# B0614) for 90 min at RT and visualized by Clarity ECL (Enhanced Chemiluminescence, Biorad, Hercules, CA, USA). All primary antibodies were diluted 1:1000 except GAPDH 1:20,000 while HRP-linked secondary antibodies diluted 1:2000 in PBS-0.1% Tween 20–5% non-fat dry milk (Bio-Rad Laboratories, Hercules, CA, USA). Images were acquired with a Biorad Chemidoc imager. Densitometric analysis was performed by using Biorad Image Lab software (Version 6.0.0, Image Lab Software, RRID:SCR_014210, Hercules, CA, USA).

### 4.16. Cell and Tissue Immunohistochemistry and Immunofluorescence

Control and siAGC1 Oli-Neu cells, as well as AGC1^+/+^ and AGC1^+/−^ neurospheres were fixed with 4% PFA for 20 min in PBS 0.1%, pH 7.4 and then washed in phosphate-buffered saline (PBS). Cells were permeabilized in PBS-0.1% Triton and aspecific sites were blocked with PBS-0.1% Triton, 5% normal goat serum (Sigma-Aldrich) for 60 min at RT. Cells were incubated overnight at 4 °C with the primary antibody, anti-Olig2 (Santa Cruz Biotechnology Cat# sc-48817, RRID:AB_2157550, Lot# C1413, 1:500 dilution), anti-NG2 (Abcam Cat# ab83178, RRID:AB_10672215, Lot# GR3194358-1, 1:500 dilution), anti-PDGFRα (Santa Cruz Biotechnology Cat# sc-338, RRID:AB_631064, Lot# F1614, 1:500 dilution), anti-PDGFα (Santa Cruz Biotechnology Cat# sc-7958, RRID:AB_2161914, Lot# C0204, 1:500 dilution), anti-TGFβR2 (Santa Cruz Biotechnology Cat# sc-17792, RRID:AB_628349, Lot# D1813, 1:500 dilution), anti-CNPase (Cell Signalling Technology Cat# 5664P, RRID:AB_10705455, Lot# 2, 1:500 dilution), anti-TGFβR1 (Santa Cruz Biotechnology Cat# sc-398, RRID:AB_632493, Lot# G0314, 1:500 dilution), anti-Ki67 (Abcam Cat# ab15580, RRID:AB_443209, Lot# GR239828-1, 1:500 dilution), anti- DCX (Abcam Cat# ab18723, RRID:AB_732011, Lot# GR324492-1, 1:500 dilution), anti-GFAP (Dakopatts Cat# Z0334, RRID:AB_10013382, Lot# 119, 1:500 dilution), anti-BrdU (Abcam Cat# ab6326, RRID:AB_305426, Lot# GR251710-3; 1:500 dilution) in PBS-0.1% Triton + 2% normal goat serum. Cells were then incubated with the secondary antibody anti-mouse tetramethyl rhodamine isothiocyanate conjugate, 1:500 dilution (Sigma-Aldrich Cat# T5393, RRID:AB_261699); anti-rabbit Alexa-488, 1:1000 dilution (Abcam Cat# ab150077, RRID:AB_2630356, Lot# GR322463-1); anti-rabbit Alexa-555, 1:1000 dilution (Abcam Cat# ab150078, RRID: AB_2722519 Lot# GR3180320-1) for 1 h and 30 min at RT. Nuclei were stained with Hoechst 33258 (2 µg/mL; Sigma-Aldrich) for 5 min. After a quick wash in PBS, Pro Long Gold Antifade Reagent (Life Technologies) was used to mount fixed and stained cells.

Forty µm brain slices were obtained by using a tissue cryostat. For immunohistochemistry with DAB staining, brain slices were washed once for 10 min in PBS, then incubated for 30 min in 0.3% H_2_O_2_ in methanol. Brain slices were then washed 3 × 10 min in PBS and then 3 × 10 min in PBS-0.1% Triton (Sigma-Aldrich). Aspecific sites were blocked by incubating brain slices for 1 h in blocking buffer (PBS-0.1% Triton + 2% normal goat serum). Brain slices were then incubated overnight with rabbit anti-Olig2 (1:500; Santa Cruz Biotechnology Cat# sc-48817, RRID:AB_2157550, Lot# C1413), rabbit anti-DCX (1:500; Abcam Cat# ab18723, RRID:AB_732011, Lot# GR324492-1) and rabbit anti-GFAP (1:2000; Dakopatts, Cat# Z0334, RRID:AB_10013382, Lot# 119) primary antibodies diluted in blocking buffer. The next day, brain slices were washed 3 × 10 min in PBS-0.1% Triton and then incubated for 2 h with diluted secondary antibodies (1:500, Santa Cruz Biotechnology Cat# sc-2004, RRID:AB_631746, Lot# D1216) in blocking buffer. Brain sections were then washed 2 × 10 min in PBS-0.1% Triton and 1 × 10 min in 50 mM Tris, pH 7.6. Brain sections were then incubated with diaminobenzidine (DAB) (Vector Laboratories Cat# SK-4100, RRID:AB_2336382), following the manufacturer’s instructions (Vector Laboratories, Burlingame, CA, USA) for 30–180 s and then promptly washed 3 × 10 min in H_2_O. Brain sections were mounted on gelatin-coated glass slides and air-dried overnight. The next day, brain sections were dehydrated 1 x 1 min in 90% ethanol and 2 × 1 min in 100% ethanol. Brain slices were then incubated for 1 min in xylene and mounted with DPX (Sigma-Aldrich). Glass slides were allowed to dry overnight.

For brain section immunofluorescence, 40 μm thick sections were used as well. Previously fixed brain sections were washed 3 × 10 min in PBS and then 3 × 10 min in PBS-0.1% Triton. Aspecific sites were then blocked by incubating brain slices for 1 h in blocking buffer (PBS-0.1% Triton + 5% normal goat serum). Brain slices were incubated overnight with rabbit anti-Olig2 (1:500; Santa Cruz Biotechnology Cat# sc-48817, RRID:AB_2157550, Lot# C1413), rabbit anti-DCX (1:500; Abcam Cat# ab18723, RRID:AB_732011, Lot# GR324492-1) rabbit anti-GFAP (1:2000; Dakopatts, Cat# Z0334, RRID:AB_10013382, Lot# 119) primary antibodies diluted in blocking buffer (PBS-0.1% Triton + 2% normal goat serum). The next day brain sections were washed 3 x 10 min in PBS-0.1% Triton and then incubated for 2 h with diluted secondary antibodies anti-rabbit Alexa-488, 1:1000 (Abcam Cat# ab150077, RRID:AB_2630356, Lot# GR322463-1); anti-mouse Alexa-555, 1:1000 (Abcam Cat# ab150114, RRID:AB_2687594, Lot# GR3173748-1) in blocking buffer. Brain sections were then washed 3 × 10 min in PBS-0.1% Triton, 10 min in PBS and then incubated for 5 min in Hoechst 33258 (2 µg/mL; Sigma-Aldrich). Brain sections were then washed 5 min in PBS and coverslips were mounted with Ultra Cruz mounting medium (Santa Cruz, Dallas, TX, USA), sealed with nail polish and air-dried for 10 min before being stored at 4 °C in the dark until used.

### 4.17. Cell Counting after Immunohistochemistry and Immunofluorescence

Stained Oli-Neu cells were photographed with a fluorescence microscope (Eclipse Hoechst staining TE 2000-S microscope; Nikon, Tokyo, Japan) while for AGC1^+/+^ and AGC1^+/−^ neurospheres and brain section, images were acquired by using a Nikon EZ-C1 confocal microscope with a 10X or 40X objective by using the z-stack function and setting 512 steps at a stack thickness of 1 μm (40 total stacks). After image acquisition, 3D image reconstruction was performed by using the z-project plugin in Fiji ImageJ2 software (Fiji, RRID:SCR_002285) and selecting the sum function.

For Tissue section images, AGC1^+/+^ and AGC1^+/−^ matched slice images were selected considering always the same rectangular area of corpus callosum (CC) and subventricular zone (SVZ) (400 µm × 161 µm). Cell volume was determined by considering a 40 µm thickness for brain sections under study and stained cell number was expressed as cells/ µm^3^. All positive cells were counted by using the manual cell counter plugin of Fiji ImageJ2 software (Fiji, RRID:SCR_002285). Labelling index was expressed as the ratio of positive/total cells; total nuclei were stained with Hoechst.

### 4.18. Statistical Analysis

All results were subject to statistical analysis with Student’s T-test or one-way and two-way ANOVA followed by Bonferroni’s *post-hoc* comparison test in order to evaluate the significance of the observed differences by using Graph Pad Prism 4 software (GraphPad Prism, RRID:SCR_002798, San Diego, CA, USA).

## 5. Conclusions

In conclusion, in the present study we demonstrate for the first time that alterations in AGC1 activity lead to deficits in OPC proliferation, due to dysregulation in the PDGFα and TGFβ pathways. These defects have been demonstrated in vitro and confirmed in our in vivo model, as well as in neurospheres. OPC proliferation defects could result in an insufficient number of precursor cells able to replace aged oligodendrocytes and in parallel premature differentiation of OPCs, further suggesting that in AGC1 deficiency there may be no defects in mature oligodendrocytes leading to hypomyelination. Therefore, deficits in OPC proliferation rather than OPC differentiation may represent a new biological target together with reduced growth and NAA synthesis in neurons to counteract a reduced myelinating filament, not only in AGC1 deficiency but also in other developmental and adult demyelinating diseases.

## Supplementary Materials

Supplementary materials can be found at https://www.mdpi.com/1422-0067/20/18/4486/s1.

## Abbreviations

AEQ	aequorin
AGC1	mitochondrial aspartate-glutamate carrier isoform 1
BrdU	Bromodeoxyuridine
CNPase	2′,3′-Cyclic-nucleotide 3′-phosphodiesterase
CTLN2	type II citrullinemia
db-cAMP	N6,2′-o-dibutyryl)-adenosine-3′,5′-mono-phosphate
DCX	doublecortin
FCCP	Carbonyl cyanide-4-(trifluoromethoxy)phenylhydrazone
GFAP	Glial fibrillary acidic protein
HBSS	Hank’s Balanced Salt Solution
MAG	myelin associated glycoprotein
MBP	myelin basic protein
NAA	n-acetyl-aspartate
NICCD	neonatal intrahepatic cholestasis
NSCs	neural stem cells
OPCs	oligodendrocyte precursors cells
PDGFα	Platelet-Derived Growth Factor α
PBS	Phosphate Buffer Solution
PFA	paraformaldehyde
RT	room temperature
SLC25A12	solute carrier family 25, member 12
SVZ	subventricular zone
TGFβ	Transforming Growth Factor β
TMRM	tetramethyl rhodamine methyl ester

## References

[1] Wibom R., Lasorsa F.M., Tohonen V., Barbaro M., Sterky F.H., Kucinski T., Naess K., Jonsson M., Pierri C.L., Palmieri F. (2009). AGC1 Deficiency Associated with Global Cerebral Hypomyelination. New Engl. J. Med..

[2] Falk M.J., Li D., Gai X., McCormick E., Place E., Lasorsa F.M., Otieno F.G., Hou C., Kim C.E., Abdel-Magid N. (2014). AGC1 Deficiency Causes Infantile Epilepsy, Abnormal Myelination and Reduced N-Acetylaspartate. JIMD Rep..

[3] Palmieri F. (2014). Mitochondrial transporters of the SLC25 family and associated diseases: A review. J. Inherit. Metab. Dis..

[4] Indiveri C., Krämer R., Palmieri F. (1987). Reconstitution of the malate/aspartate shuttle from mitochondria. J. Boil. Chem..

[5] Palmieri L., Pardo B., Lasorsa F.M., del Arco A., Kobayashi K., Iijima M., Runswick M.J., Walker J.E., Saheki T., Satrústegui J. (2001). Citrin and aralar1 are Ca(2+)-stimulated aspartate/glutamate transporters in mitochondria. EMBO J..

[6] Del Arco A., Satrústegui J. (1998). Molecular Cloning of Aralar, a New Member of the Mitochondrial Carrier Superfamily That Binds Calcium and Is Present in Human Muscle and Brain. J. Boil. Chem..

[7] Saheki T., Kobayashi K. (2002). Mitochondrial aspartate glutamate carrier (citrin) deficiency as the cause of adult-onset type II citrullinemia (CTLN2) and idiopathic neonatal hepatitis (NICCD). J. Hum. Genet..

[8] Ledeen R.W., Wang J., Wu G., Lu Z.H., Chakraborty G., Meyenhofer M., Tyring S.K., Matalon R. (2006). Physiological role of N-acetylaspartate: Contribution to myelinogenesis. Adv. Exp. Med. Biol..

[9] Jalil M.A., Begum L., Contreras L., Pardo B., Iijima M., Li M.X., Ramos M., Marmol P., Horiuchi M., Shimotsu K. (2005). ReducedN-Acetylaspartate Levels in Mice Lacking Aralar, a Brain- and Muscle-type Mitochondrial Aspartate-glutamate Carrier. J. Boil. Chem..

[10] Ramos M., Pardo B., Saheki T., Del Arco A., Satrústegui J., Llorente-Folch I., Llorente-Folch I. (2011). Deficiency of the mitochondrial transporter of aspartate/glutamate aralar/AGC1 causes hypomyelination and neuronal defects unrelated to myelin deficits in mouse brain. J. Neurosci. Res..

[11] Gómez-Galán M., Makarova J., Llorente-Folch I., Saheki T., Pardo B., Satrústegui J., Herreras O. (2012). Altered postnatal development of cortico-hippocampal neuronal electric activity in mice deficient for the mitochondrial aspartate-glutamate transporter. J. Cereb. Blood Flow Metab..

[12] Llorente-Folch I., Rueda C.B., Amigo I., Del Arco A., Saheki T., Pardo B., Satrustegui J. (2013). Calcium-Regulation of Mitochondrial Respiration Maintains ATP Homeostasis and Requires ARALAR/AGC1-Malate Aspartate Shuttle in Intact Cortical Neurons. J. Neurosci..

[13] Llorente-Folch I., Sahún I., Contreras L., Casarejos M.J., Grau J.M., Saheki T., Mena M.A., Satrústegui J., Dierssen M., Pardo B. (2013). AGC1-malate aspartate shuttle activity is critical for dopamine handling in the nigrostriatal pathway. J. Neurochem..

[14] Contreras L., Ramirez L., Du J., Hurley J.B., Satrústegui J., De La Villa P. (2016). Deficient glucose and glutamine metabolism in Aralar/AGC1/Slc25a12 knockout mice contributes to altered visual function. Mol. Vis..

[15] Juaristi I., García-Martín M.L., Satrústegui J., Llorente-Folch I., Pardo B., Rodrigues T.B. (2017). ARALAR/AGC1 deficiency, a neurodevelopmental disorder with severe impairment of neuronal mitochondrial respiration, does not produce a primary increase in brain lactate. J. Neurochem..

[16] Boulanger J., Messier C. (2014). From precursors to myelinating oligodendrocytes: Contribution of intrinsic and extrinsic factors to white matter plasticity in the adult brain. Neuroscience.

[17] Chamberlain K.A., Nanescu S.E., Psachoulia K., Huang J.K. (2016). Oligodendrocyte regeneration: Its significance in myelin replacement and neuroprotection in multiple sclerosis. Neuropharmacol.

[18] Sakurai T., Ramoz N., Barreto M., Gazdoiu M., Takahashi N., Gertner M., Dorr N., Gama Sosa M.A., De Gasperi R., Perez G. (2010). Slc25a12 disruption alters myelination and neurofilaments: A model for a hypomyelination syndrome and childhood neurodevelopmental disorders. Biol. Psychiatry.

[19] Profilo E., Peña-Altamira L.E., Corricelli M., Castegna A., Danese A., Agrimi G., Petralla S., Giannuzzi G., Porcelli V., Sbano L. (2017). Down-regulation of the mitochondrial aspartate-glutamate carrier isoform 1 AGC1 inhibits proliferation and N-acetylaspartate synthesis in Neuro2A cells. Biochim. Biophys. Acta (BBA)-Mol. Basis Dis..

[20] Belousov V.V., Fradkov A.F., Lukyanov K.A., Staroverov D.B., Shakhbazov K.S., Terskikh A.V., Lukyanov S. (2006). Genetically encoded fluorescent indicator for intracellular hydrogen peroxide. Nat. Methods.

[21] Hendzel M.J., Wei Y., Mancini M.A., Van Hooser A., Ranalli T., Brinkley B.R., Bazett-Jones D.P., Allis C.D. (1997). Mitosis-specific phosphorylation of histone H3 initiates primarily within pericentromeric heterochromatin during G2 and spreads in an ordered fashion coincident with mitotic chromosome condensation. Chromosom.

[22] Takebayashi H., Nabeshima Y., Yoshida S., Chisaka O., Ikenaka K., Nabeshima Y.-I. (2002). The Basic Helix-Loop-Helix Factor Olig2 Is Essential for the Development of Motoneuron and Oligodendrocyte Lineages. Curr. Boil..

[23] Francis F., Koulakoff A., Boucher D., Chafey P., Schaar B., Vinet M.-C., Friocourt G., McDonnell N., Reiner O., Kahn A. (1999). Doublecortin Is a Developmentally Regulated, Microtubule-Associated Protein Expressed in Migrating and Differentiating Neurons. Neuron.

[24] Brunner C., Lassmann H., Waehneldt T.V., Matthieu J., Linington C. (1989). Differential Ultrastructural Localization of Myelin Basic Protein, Myelin/Oligodendroglial Glycoprotein and 2′,3′-Cyclic Nucleotide 3′-Phosphodiesterase in the CNS of Adult Rats. J. Neurochem..

[25] Baumann N., Pham-Dinh D. (2001). Biology of Oligodendrocyte and Myelin in the Mammalian Central Nervous System. Physiol. Rev..

[26] Accetta R., Damiano S., Morano A., Mondola P., Paternò R., Avvedimento E.V., Santillo M. (2016). Reactive Oxygen Species Derived from NOX3 and NOX5 Drive Differentiation of Human Oligodendrocytes. Front. Cell. Neurosci..

[27] Diemel L.T., Jackson S.J., Cuzner M.L. (2003). Role for TGF-β1, FGF-2 and PDGF-AA in a myelination of CNS aggregate cultures enriched with macrophages. J. Neurosci. Res..

[28] Palazuelos J., Klingener M., Aguirre A. (2014). TGFβ Signalling Regulates the Timing of CNS Myelination by Modulating Oligodendrocyte Progenitor Cell Cycle Exit through SMAD3/4/FoxO1/Sp1. J. Neurosci..

[29] Koch H.B., Zhang R., Verdoodt B., Bailey A., Zhang C.D., Yates J.R., Menssen A., Hermeking H. (2007). Large-scale identification of c-MYC-associated proteins using a combined TAP/MudPIT approach. Cell Cycle.

[30] Xie D., Shen F., He S., Chen M., Han Q., Fang M., Zeng H., Chen C., Deng Y. (2016). IL-1β induces hypomyelination in the periventricular white matter through inhibition of oligodendrocyte progenitor cell maturation via FYN/MEK/ERK signaling pathway in septic neonatal rats. Glia.

[31] Remaud S., Ortiz F.C., Perret-Jeanneret M., Aigrot M.S., Gothié J.D., Fekete C., Kvárta-Papp Z., Gereben B., Langui D., Lubetzki C. (2017). Transient hypothyroidism favors oligodendrocyte generation providing functional remyelination in the adult mouse brain. Elife.

[32] Ayanlaja A.A., Xiong Y., Gao Y., Ji G., Tang C., Abdikani A.Z., Gao D. (2017). Distinct Features of Doublecortin as a Marker of Neuronal Migration and Its Implications in Cancer Cell Mobility. Front. Mol. Neurosci..

[33] Gil-Perotín S., Duran-Moreno M., Ramirez M., García-Verdugo J.M., Gil-Perotin S., Duran-Moreno M., Cebrián-Silla A., García-Belda P., Garcia-Verdugo J.M., Gil-Perotin S. (2013). Adult Neural Stem Cells from the Subventricular Zone: A Review of the Neurosphere Assay. Anat. Rec. Adv. Integr. Anat. Evol. Boil..

[34] Walker T.L., Kempermann G. (2014). One Mouse, Two Cultures: Isolation and Culture of Adult Neural Stem Cells from the Two Neurogenic Zones of Individual Mice. J. Vis. Exp..

[35] Hart I.K., Richardson W.D., Bolsover S.R., Raff M.C. (1989). PDGF and intracellular signaling in the timing of oligodendrocyte differentiation. J. Cell Boil..

[36] Noble M., Murray K., Stroobant P., Waterfield M.D., Riddle P. (1988). Platelet-derived growth factor promotes division and motility and inhibits premature differentiation of the oligodendrocyte/type-2 astrocyte progenitor ceil. Nature.

[37] Raff M.C., Lillien L.E., Richardson W.D., Burne J.F., Noble M.D. (1988). Platelet-derived growth factor from astrocytes drives the clock that times oligodendrocyte development in culture. Nature.

[38] Dahlin M., Mártin D.A., Hedlund Z., Jönsson M., Von Döbeln U., Wedell A. (2015). The ketogenic diet compensates for AGC1 deficiency and improves myelination. Epilepsia.

[39] Heo G., Kim S.H., Chang M.J. (2017). Effect of ketogenic diet and other dietary therapies on anti-epileptic drug concentrations in patients with epilepsy. J. Clin. Pharm. Ther..

[40] Barañano K.W., Hartman A.L. (2008). The Ketogenic Diet: Uses in Epilepsy and Other Neurologic Illnesses. Curr. Treat. Options Neurol..

[41] Vozza A., Parisi G., De Leonardis F., Lasorsa F.M., Castegna A., Amorese D., Marmo R., Calcagnile V.M., Palmieri L., Ricquier D. (2014). UCP2 transports C4 metabolites out of mitochondria, regulating glucose and glutamine oxidation. Proc. Natl. Acad. Sci. USA.

[42] Lasorsa F.M., Pinton P., Palmieri L., Fiermonte G., Rizzuto R., Palmieri F. (2003). Recombinant expression of the Ca(2+)-sensitive aspartate/glutamate carrier increases mitochondrial ATP production in agonist-stimulated Chinese hamster ovary cells. J. Biol. Chem..

[43] Bonora M., Giorgi C., Bononi A., Marchi S., Patergnani S., Rimessi A., Rizzuto R., Pinton P. (2013). Subcellular calcium measurements in mammalian cells using jellyfish photoprotein aequorin-based probes. Nat. Protoc..

[44] Suski J.M., Lebiedzinska M., Bonora M., Pinton P., Duszynski J., Wieckowski M.R. (2012). Relation Between Mitochondrial Membrane Potential and ROS Formation. Mitochondrial Bioenerg..

[45] Grove B.D., Bruchey A.K. (2001). Intracellular Distribution of Gravin, a PKA and PKC Binding Protein, in Vascular Endothelial Cells. J. Vasc. Res..

[46] Lowry O.H., Rosebrough N.J., Farr A.L., Randall R.J. (1951). Protein measurement with the Folin phenol reagent. J. Boil. Chem..

[47] Goubert E., Mircheva Y., Lasorsa F.M., Melon C., Profilo E., Sutera J., Becq H., Palmieri F., Palmieri L., Aniksztejn L. (2017). Inhibition of the Mitochondrial Glutamate Carrier SLC25A22 in Astrocytes Leads to Intracellular Glutamate Accumulation. Front. Cell. Neurosci..

[48] Choudhry P. (2016). High-Throughput Method for Automated Colony and Cell Counting by Digital Image Analysis Based on Edge Detection. PLoS ONE.

