# Supplementary material for: Deficiency of Mitochondrial Aspartate-Glutamate Carrier 1 Leads to Oligodendrocyte Precursor Cell Proliferation Defects Both In Vitro and In Vivo
Source: Int J Mol Sci. 2019 Sep 11;20(18):4486. doi: 10.3390/ijms20184486 (PMC6769484; doi:10.3390/ijms20184486)
Supplement: Supplementary file 1 [file ijms-20-04486-s001.pdf]

Deficiency of Mitochondrial Aspartate-Glutamate Carrier 1 Leads to Oligodendrocyte Precursor Cell Proliferation Defects Both *in vitro* and *in vivo*

Sabrina Petralla, Luis Emiliano Peña-Altamira, Eleonora Poeta, Francesca Massenzio, Marco Virgili, Simona Nicole Barile, Luigi Sbano, Emanuela Profilo, Mariangela Corricelli, Alberto Danese, Carlotta Giorgi, Rita Ostan, Miriam Capri, Paolo Pinton, Ferdinando Palmieri, Francesco Massimo Lasorsa and Barbara Monti

1. Oli-Neu Cell Differentiation

Control and siAGC1 Oli-Neu cells were plated at a density of 1 × 10^5 on 60-mm diameter poly-L-lysine (10 μg/mL) coated Petri dishes (Corning Incorporated, NY, USA) with SATO medium supplemented with 1% HS and 1 mM dibutyril-cAMP (db-cAMP, Sigma-Aldrich). Cells were allowed to differentiate for 3 days with no medium change. After 0, 24, 48 and 72 h from plating, images from 5 randomly selected fields for each Petri dish were acquired with an Eclipse TS100 (Nikon) microscope by using a 10X objective.

2. Cell Sample Preparation for Western Blot Analysis

Whole cell lysate samples were obtained by washing Oli-Neu cell cultures once with PBS and resuspending cell cultures in cell lysis buffer (50 mM Tris pH 7.4, 1 mM EDTA, 1% SDS, 10 μl/mL protease and phosphatase inhibitor cocktails). Samples were sonicated with a Branson 250 digital sonifier for 3 pulses of 2 s each (waiting for 5 s between each pulse) at 10% power output and stored at −80 °C until used. Total protein sample content was determined by using the Lowry quantification method [46] and 30 μg of each sample with Laemli loading buffer (1M Tris-HCl pH 6.8; 20% sodium dodecyl sulfate; 0.4 μl/mL glycerol; 2 g/L bromophenol blue and 2M dithiothreitol; all from Sigma-Aldrich), were loaded per lane for western blot analysis.

3. Western Blotting

Cell samples obtained as described, were briefly sonicated and resolved in SDS-PAGE (Sodium Dodecyl Sulfate - PolyAcrylamide Gel Electrophoresis), before electroblotting. Membranes were incubated with antibodies against AGC1 (1:1000, Santa Cruz Biotechnology Cat# sc-271056, RRID:AB_10608837, Lot# L1312), MAG (1:1000, Santa Cruz Biotechnology Cat# sc-15324, RRID:AB_670104, Lot# C1910) and GAPDH (1:20000, Santa Cruz Biotechnology Cat# sc-32233, RRID:AB_627679, Lot# B0514) for Oli-Neu cells and against NG2 (1:1000, Abcam Cat# ab83178, RRID:AB_10672215, Lot# GR3194358-1), Olig2 (1:1000, Santa Cruz Biotechnology Cat# sc-48817, RRID:AB_2157550, Lot# C1413), CNPase (1:1000, Cell Signaling Technology Cat# 5664P, RRID:AB_10705455, Lot# 2), DCX (1:1000, Abcam Cat# ab18723, RRID:AB_732011, Lot# GR324492-1), GFAP (1:2000, Dakopatts Cat# Z0334, RRID:AB_10013382, Lot# 119) for neurosphere, all diluted in PBS-0.1% Tween 20 – 5% non-fat dry milk (Bio-Rad Laboratories, Hercules, CA, USA), then with HRP-linked secondary antibodies goat anti-rabbit (Santa Cruz Biotechnology Cat# sc-2004, RRID:AB_631746, Lot# D1216), goat anti-mouse (Santa Cruz Biotechnology Cat# sc-2005, RRID:AB_631736, Lot# B1616), donkey anti-goat (Santa Cruz Biotechnology Cat# sc-2020, RRID:AB_631728, Lot# B0614), diluted 1:2000 in PBS-0.1% Tween and visualized by Clarity ECL (Enhanced ChemiLuminescence; Biorad, USA). Images were acquired with a Biorad Chemidoc imager. Densitometric analysis was performed by using Biorad Image Lab software (Version 6.0.0) (Image Lab Software, RRID:SCR_014210).

4. Oli-Neu Cell Cycle Analysis by Flow Cytometry

Cell cycle analysis was performed according to Nüsse [1]. Briefly, 70% confluent Oli-Neu Petri dishes were passaged and 2 × 10^6 Oli-Neu cells per sample were centrifuged at 300× *g* for 10 min. Cell cycle analysis was performed by the method of 2bromo-5deoxyuridine (BrdU) incorporation and propidium iodide (PI) staining, as previously described [2]. Briefly, 70% confluent Oli-Neu Petri dishes were previously labeled with BrdU for 30 min and passaged and 2 × 10^6 Oli-Neu cells per sample were centrifuged at 300× *g* for 10 min, washed two times with 1ml of PBS solution containing 0.5% Tween 20, and resuspended in 1ml of HCl 2N. After a 30 min incubation at RT, cells were spun down at 300 g for 1 min, washed once in 0.1M Borax, and resuspended in 200 µl of diluted anti-BrdU mouse mAb (BD, San José, CA). Cells were incubated for 60 min at 4 °C, washed twice and resuspended in 200 µl of diluted secondary antibody (goat-anti-mouse IgG-FITC conjugate, BD, San José, CA). After a 30 min incubation at 4 °C cells were washed two times and resuspended in 200 µl of PBS solution with 0.5% Tween 20 and 200 µl of PI working solution (PI 50 mg/mL in 3.4 mM trisodium citrate, 9.65 mM NaCl plus 0.03% Nonidet P40). After 15 min at 4°C in the dark cells were analyzed with the FACScalibur flow cytometer (BD, San Josè, CA, USA) equipped with a single 488 nm argon laser and analyzed by Cell Quest software (BD).

5. Analysis of Mitochondrial Morphology in Control and siAGC1 Oli-Neu Cells

The mitochondrial morphology of Control Oli-Neu (left panel and white columns) or siAGC1 Oli-Neu (right panel and black columns) cells was analyzed by loading the cells with 100 nM tetramethyl rhodamine methyl ester (TMRM) for 30 min at 37 °C. The images were taken by confocal microscopy (see methods section). The mitochondrial network, described in number and volume, and 3D renders were obtained with Imaris 4.0 (Bitplane) (Imaris, RRID:SCR_007370).

6. Tissue Immunohistochemistry

Forty µm brain slices were obtained by using a tissue cryostat. For immunohistochemistry with DAB staining, brain slices were washed once for 10 min in PBS, then incubated for 30 min in 0.3% H_2O_2 in methanol. Brain slices were then washed 3 × 10 min in PBS and then 3 × 10 min in PBS-0.1% Triton (Sigma-Aldrich). Aspecific sites were blocked by incubating brain slices for 1 h in blocking buffer (PBS-0.1% Triton + 2% normal goat serum [Sigma-Aldrich]). Brain slices were then incubated overnight with rabbit anti-pH3 serine 10 (1:500; Santa Cruz Biotechnology Cat# sc-8656-R, RRID:AB_653256, Lot# B1909), rabbit anti-CNPase (1:500; Cell Signaling Technology Cat# 5664P, RRID:AB_10705455, Lot# 2), rabbit anti-MBP (1:500; Santa Cruz Biotechnology Cat# sc-271524, RRID:AB_10655672, Lot# A2017) primary antibodies diluted in blocking buffer. The next day, brain slices were washed 3 × 10 min in PBS-0.1% Triton and then incubated for 2 h with diluted secondary antibodies (1:500, Santa Cruz Biotechnology Cat# sc-2004, RRID:AB_631746, Lot# D1216) in blocking buffer. Brain sections were then washed 2 × 10 min in PBS-0.1% Triton and 1 × 10 min in 50 mM Tris, pH 7.6. Brain sections were then incubated with diaminobenzydine (DAB) (Vector Laboratories Cat# SK-4100, RRID:AB_2336382), following the manufacturer's instructions (Vector Laboratories, Burlingame, CA, USA) for 30–180 s and then promptly washed 3 × 10 min in H_2O. Brain sections were mounted on gelatin-coated glass slides and air-dried overnight. The next day, brain sections were dehydrated 1 × 1 min in 90% ethanol and 2 × 1 min in 100% ethanol. Brain slices were then incubated for 1 min in xylene and mounted with DPX (Sigma-Aldrich). Glass slides were allowed to dry overnight. p-$H3^+$ cells were counted with Fiji ImageJ2 software (Fiji, RRID:SCR_002285) as follows: $AGC1^{+/+}$ and $AGC1^{+/-}$ matched slice images were selected. The same rectangular area (400 × 161 µm) was considered for all section images. DAB labeled cells were counted by using the manual cell counter plugin. Cell volume was determined by considering a 40 µm thickness for brain sections under study and $Olig2^+$ cell number was expressed as cells/μm^3. For immunofluorescence, previously fixed brain slices were washed 3 × 10 min in PBS-0.1% Triton (Sigma-Aldrich). Aspecific sites were blocked by incubating brain slices for 1 h in blocking buffer (PBS-0.1% Triton + 10% normal goat serum [Sigma-Aldrich]). Brain slices were then incubated overnight rabbit anti-NG2 (1:500; Abcam Cat# ab83178, RRID:AB_10672215, Lot# GR3194358-1) diluted in PBS-0.1% Triton + 5% normal goat

serum. The next day, brain slices were washed 3 × 10 min in PBS-0.1% Triton and then incubated for 2 h with diluted secondary antibodies anti-rabbit Alexa-555, 1:1000, (Abcam Cat# ab150078, RRID: AB_2722519 Lot# GR3180320-1) in PBS-0.1% Triton + 5% normal goat serum. Slices were then washed 3 × 10 min in PBS-0.1% Triton, 10 min in PBS, incubated for 5 min in Hoechst 33258 (2 µg/mL; Sigma-Aldrich), washed again 5 min in PBS and coverslips were mounted with Ultra Cruz mounting medium (Santa Cruz), sealed with nail polish and air-dried for 10 min before being stored at 4 °C in the dark until used.

7. Measure of CNPase Activity

CNPase (2 ′, 3′ cyclic nucleotide, 3 ′phosphohydrolase) is a myelin-membrane associated enzyme [24] used as an oligodendrocyte and myelination marker. CNPase activity was determined by using a colorimetric reaction resulting in the formation of a ferrous salt reduced complex between molybdate and inorganic phosphate [3]. Briefly, samples were prepared by adding 5 µL of AGC1$^{+/+}$ and AGC1$^{+/-}$ mouse brain homogenate and 195 µL of substrate (2 ′3′ cyclic-AMP, 7.7 mM Tris/50 mM Maleate pH 6.2); and samples were shortly vortexed. Samples were then incubated at 30°C for 20 min after which the reaction was blocked by incubating samples at 100 °C for 1 min and then at 30 °C. 100 µl of alkaline phosphatase solution (0.7 mg/mL diluted in 300 mM Tris HCl – 1 mM $MgCl_2$, pH 9.0) were added to each sample in order to allow adenosine monophosphate hydrolysis; the reaction was blocked after 45 min by adding 900 µL of 10% trichloroacetic acid (TCA) to samples and samples were then centrifuged at 3500× *rpm* for 10 min. 700 µL of supernatant were transferred into a microcentrifuge tube and 500 µL of reagent ($FeSO_4$ * 7 H_2O pm 278.02 + molybdate reagent + H_2O) were added and then vortexed. The colorimetric reaction was developed after 2/3 min at 30 °C. A standard calibration curve was set up with a 1 mM Na_2HPO_4 solution. Calibration curve and sample absorbance measurements were performed with a spectrophotometer set at 660 nm.

8. Neurospheres Differentiation

To study neurosphere differentiation, AGC1$^{+/+}$ and AGC1$^{+/-}$ SVZ-derived neurospheres were counted and plated on 13-mm coverslips (30 neurospheres/well) previously treated with poly-L-lysine (10 µg/mL) and then incubated at 37 °C for at least 3 h with fibronectin (1 µg/mL) in order to allow stem cell adhesion and subsequent differentiation. Cells were then plated in complete DMEM-F12 medium and allowed to differentiate for 7 days in an incubator at 37 °C. After differentiation, neurospheres were fixed with 4% PFA for 30 min, washed with PBS and stored at 4 °C in PBS until used for immunofluorescence analysis or resuspended in cell lysis buffer (50 mM Tris pH 7.4, 1 mM EDTA, 1% SDS, 10 µl/mL protease and phosphatase inhibitor cocktails) for western blot analysis.

9. Neurospheres Immunofluorescence

For neurospheres immunofluorescence, previously fixed neurospheres were washed 3 × 10 min in PBS and then 3 × 10 min in PBS-0.1% Triton (Sigma-Aldrich). Aspecific sites were then blocked by incubating cells for 1 h in blocking buffer (PBS-0.1% Triton + 5% normal goat serum). Neurospheres were then incubated overnight with rabbit anti-Olig2 (1:500; Santa Cruz Biotechnology Cat# sc-48817, RRID:AB_2157550, Lot# C1413), rabbit anti-CNPase (1:500; Cell Signaling Technology Cat# 5664P, RRID:AB_10705455, Lot# 2), rabbit anti-DCX (1:500; Abcam Cat# ab18723, RRID:AB_732011, Lot# GR324492-1) rabbit anti-GFAP (1:2000; Dakopatts, Cat# Z0334, RRID:AB_10013382, Lot# 119) primary antibodies diluted in PBS-0.1% Triton + 2% normal goat serum. The next day neuropheres were washed 3 × 10 min in PBS-0.1% Triton and then incubated for 2 h with diluted secondary antibodies anti-rabbit Alexa-488, 1:1000, (Abcam Cat# ab150077, RRID:AB_2630356, Lot# GR322463-1); anti-mouse Alexa-555, 1:1000 (Abcam Cat# ab150114, RRID:AB_2687594, Lot# GR3173748-1) in PBS-0.1% Triton + 2% normal goat serum. Neurospheres were then washed 3 × 10 min in PBS-0.1% Triton, 10 min in PBS, incubated for 5 min in Hoechst 33258 (2 µg/mL; Sigma-Aldrich), washed again 5 min in

PBS and coverslips were mounted with Ultra Cruz mounting medium (Santa Cruz), sealed with nail polish and air-dried for 10 min before being stored at 4 °C in the dark until used.

For AGC1$^{+/+}$ and AGC1$^{+/-}$ brain slices and neurospheres, images were acquired by using a Nikon EZ-C1 confocal microscope with a 10X or 40X objective by using the z-stack function and setting 512 steps at a stack thickness of 1 μm (40 total stacks). After image acquisition, 3D image reconstruction was performed by using the z-project plugin in Fiji ImageJ2 software (Fiji, RRID:SCR_002285) and selecting the sum function.

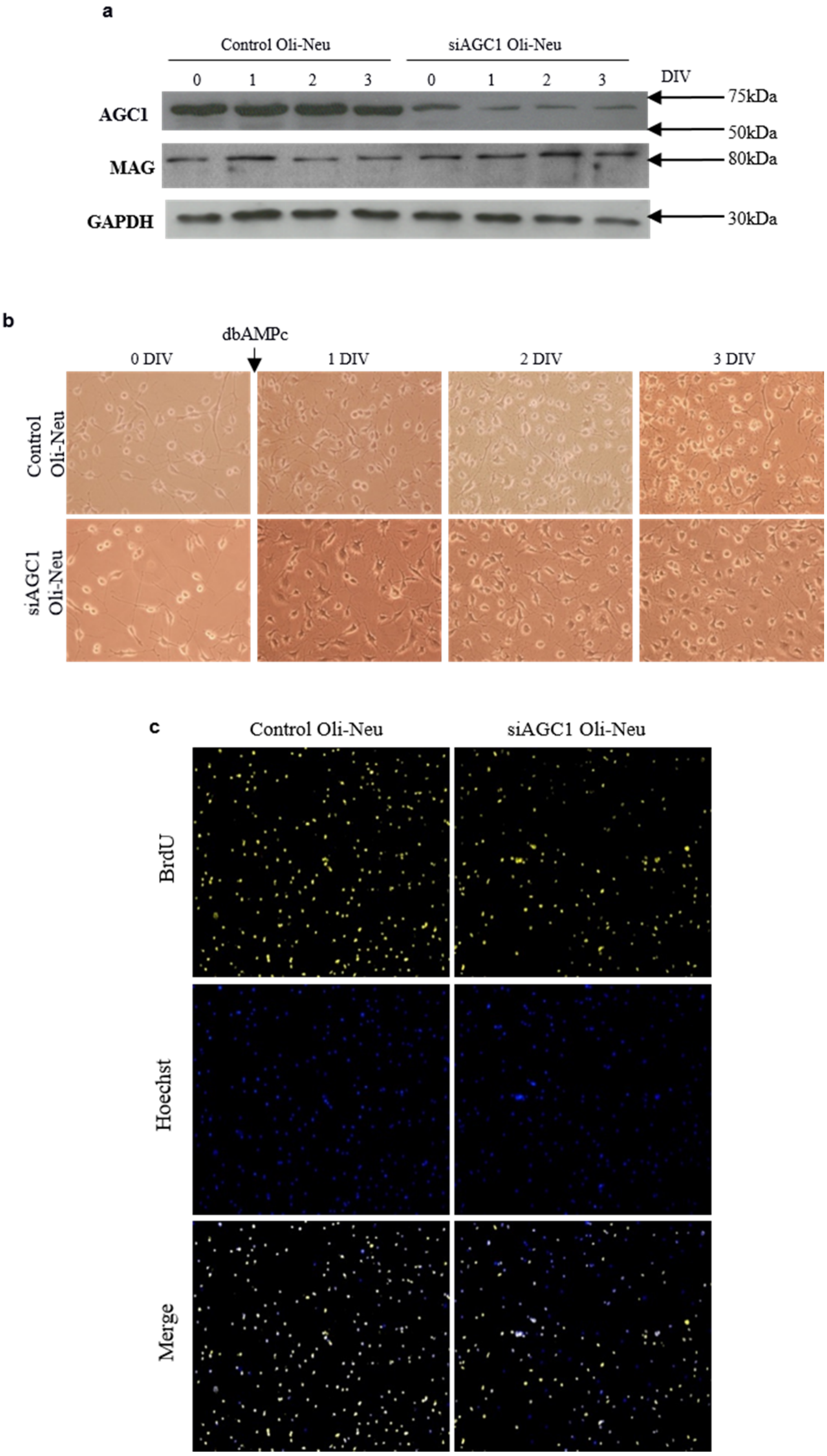

Figure. S1. No change in stimulated siAGC1 Oli-Neu cell differentiation and BrdU immunofluorescence. Western blot (**a**) and light microscopy (**b**) analysis of control and siAGC1 Oli-Neu cells induced to differentiate with 1mM db-cAMP at different days after treatment show no changes neither in AGC1 and MAG expression (using GAPDH as reference loading control) nor in Oli-Neu morphological differentiation. BrdU immunofluorescence (**c**) show a lower level of proliferating cells in siAGC1 Oli-Neu compared to control ones.

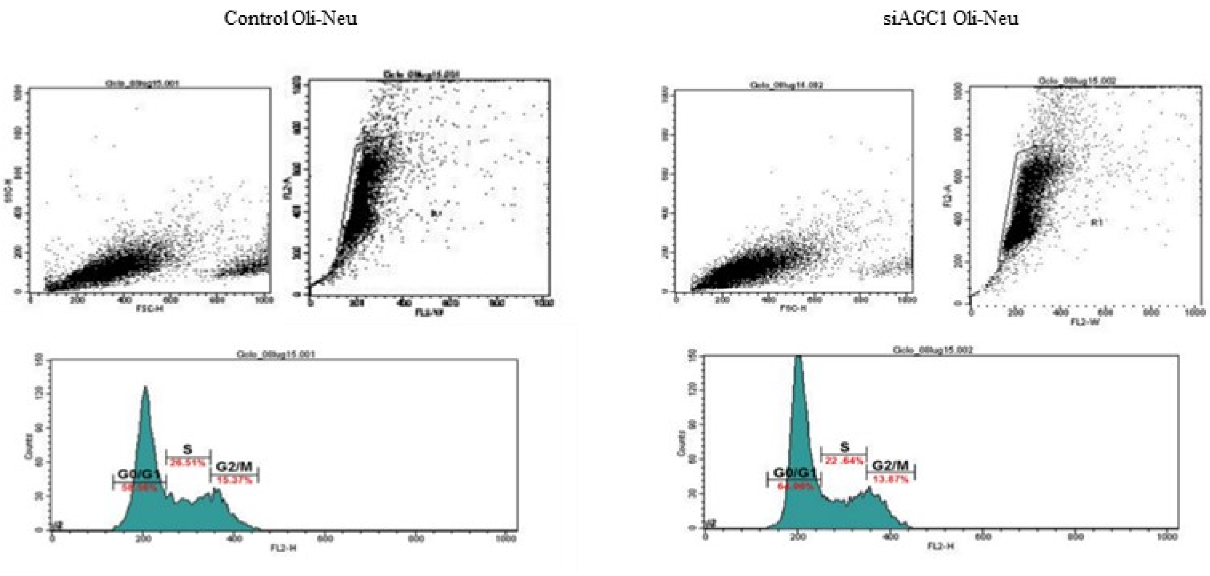

	OLI NEU control	OLI NEU siAGC1
G0-G1	58.56%	64.06%
S	26.51%	22.64%
G2/M	15.37%	13.87%

Figure S2. Flow cytometry analysis of cell cycle as DNA content (FL-2). Flow cytometry analysis of control (left panels) and siAGC1 Oli-Neu cells (right panels) shows a slightly higher number of siAGC1 Oli-Neu cells in G0/G1 phases compared to control Oli-Neu cells (64% vs. 58% respectively), whereas slight differences were observed for S phase (26% vs. 22% respectively) and for G2/M phases (15 vs. 13%). Ten thousands cell were acquired in the gate (R1) as unique experiment.

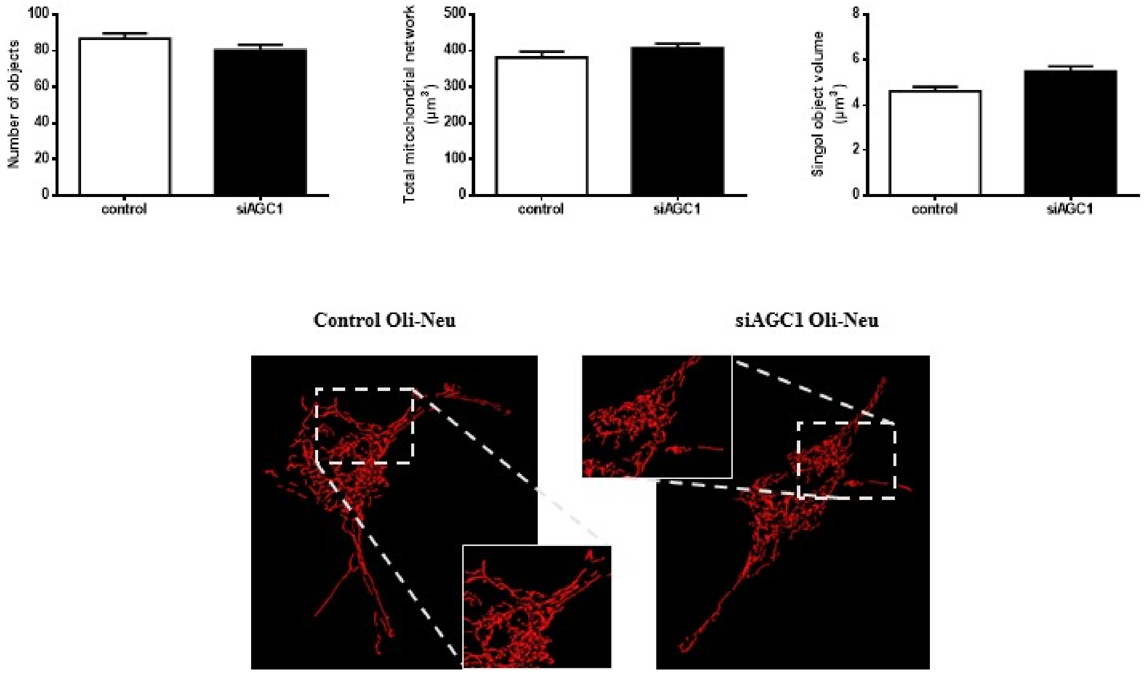

Figure S3. No change in mitochondrial morphology in siAGC1 Oli-Neu cells. The mitochondrial morphology of control Oli-Neu (white columns and left panel below) or siAGC1 Oli-Neu (black columns and right panel below) cells was analyzed by loading cells with 100 nM tetramethyl rhodamine methyl ester (TMRM) for 30 min at 37 °C. The images were taken by confocal microscopy (see Methods section). The mitochondrial network, described in number and volume, and 3D renders were obtained with Imaris 4.0 (Bitplane). Representative images and quantitative data illustrating the number of TMRM+ 3D objects per cell (means ± SEM, n = 80).

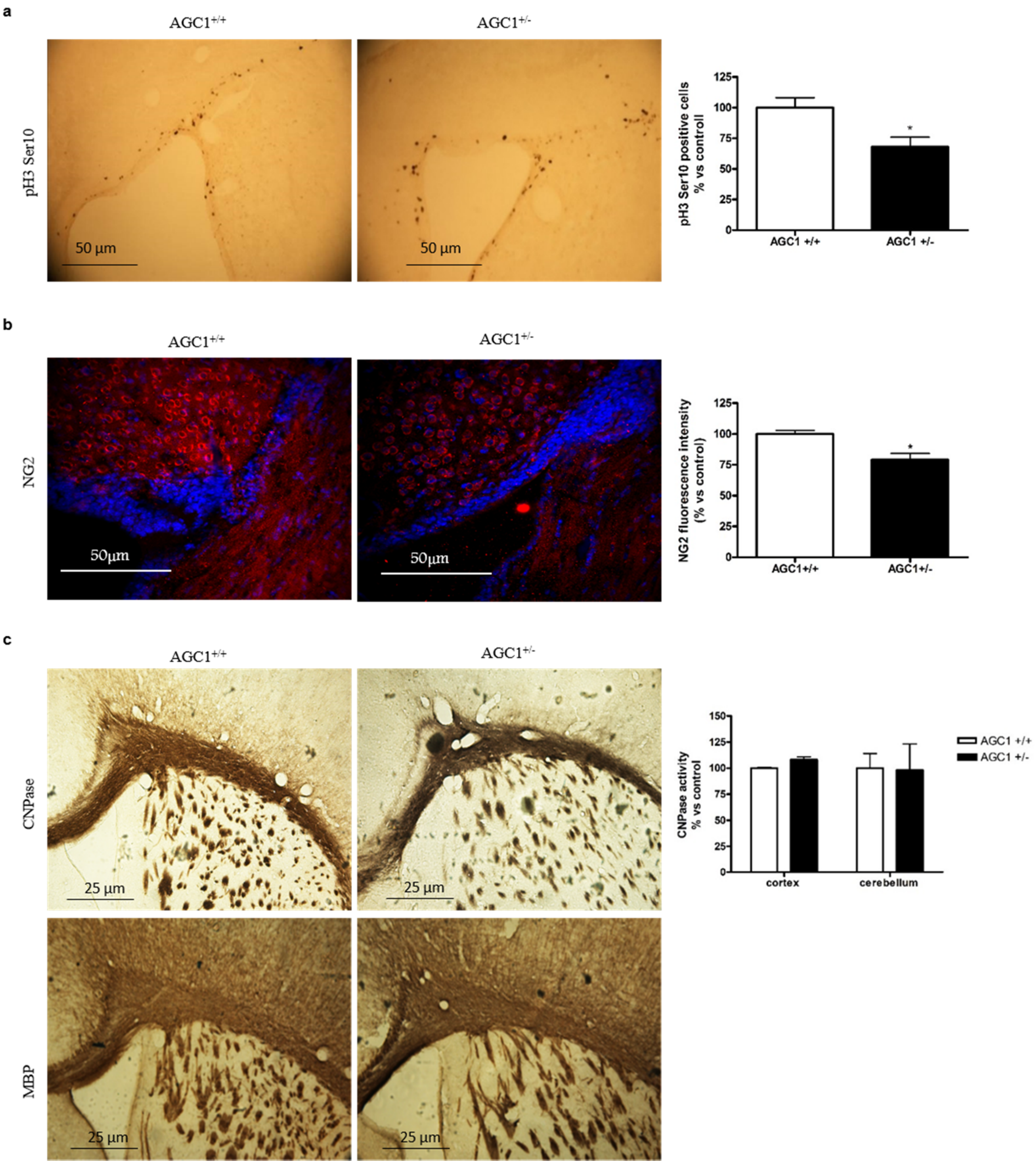

Figure S4. Proliferation deficits with no change in oligodendrocyte differentiation in 21-day old AGC1$^{+/-}$ mice. Immunohistochemical analysis and positive cell counting (**a**) of phosphorylated histone H3, markers of proliferating cells, in the corpus callosum and subventricular zone of 21 day-old AGC1$^{+/+}$ and AGC1$^{+/-}$ mice (scale bar: 50 µm) Immunofluorescence analysis and fluorescence intensity labeling (**b**) of NG2, markers of OPCs, in the corpus callosum and subventricular zone of 21 day-old AGC1$^{+/+}$ and AGC1$^{+/-}$ mice (scale bar: 50 µm). Bars are expressed as percentage vs. AGC1$^{+/+}$ and represent the mean ± SE of three experiments. * $p < 0.05$ compared to AGC1$^{+/+}$ mice, Student's t-Test. Immunohistochemical analysis of CNPase and MBP (**c**), both markers of differentiated oligodendrocytes, in the corpus callosum and subventricular zone (scale bar: 25 µm) and measurement of CNPase activity in cortex and cerebellum show no alteration in mature oligodendrocytes of 21 day-old AGC1$^{+/-}$ ($n = 8$) mice compared to AGC1$^{+/+}$ ($n = 8$) mice. Bars represent the mean ± SE of two experiments.

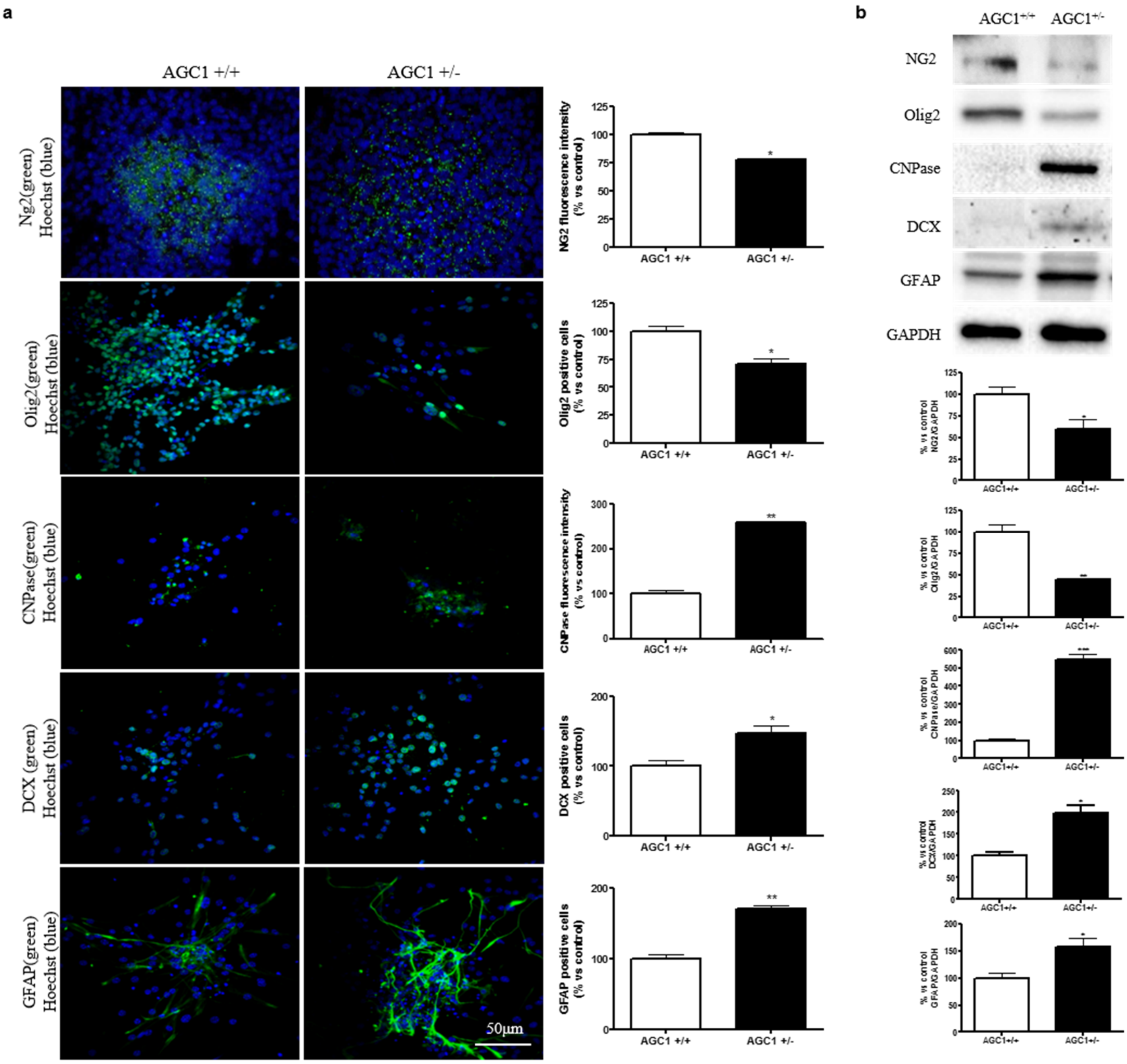

Figure S5. Spontaneous differentiation of neurospheres from the SVZ of AGC1$^{+/+}$ and AGC1$^{+/-}$ mice. AGC1$^{+/+}$ and AGC1$^{+/-}$ SVZ-derived neurospheres were plated allowed to differentiate for 7 days in at 37 °C on coverslips previously treated with poly-L-lysine and fibronectin. For immunofluorescence staining (**a**) NG2 and Olig2, CNPase, DCX and GFAP were used as specific markers for OPCs, mature oligodendrocytes, NSCs and astrocytes respectively (nuclei labeled with Hoechst). Scale bar: 50 μm. Western blot analysis and relative densitometries of NG2, Olig2, CNPase, DCX and GFAP in AGC1$^{+/+}$ and AGC1$^{+/-}$ neurospheres (**b**). Densitometry is the ratio between the expression level of each protein and GAPDH as reference loading control and is expressed as percentage vs AGC1$^{+/+}$ neurospheres. Values are the mean ± SE of 3 independent experiments performed in triplicate, * $p < 0.05$, ** $p < 0.01$, *** $p < 0.001$ compared to AGC1$^{+/+}$ neurospheres, Student's *t*-test.

References

1. Nüsse, M. Cell cycle kinetics of irradiated synchronous and asynchronous tumor cells with DNA distribution analysis and BrdUrd-Hoechst 33258-technique. *Cytom* **1981**, *2*, 70–79.
2. Barbieri, D.; Troiano, L.; Grassilli, E.; Agnesini, C.; Cristofalo, E.A.; Monti, D.; Capri, M.; Cossarizza, A.; Franceschi, C. Inhibition of apoptosis by zinc: A reappraisal. *Biochem. Biophys. Res. Commun.* **1992**, *187*, 1256–1261.

3. Prohaska, J.R.; Clark, D.A.; Wells, W.W. Improved rapidity and precision in the determination of brain 2′,3′-cyclic nucleotide 3′-phosphohydrolase. *Anal. Biochem.* **1973**, *56*, 275–282.
